# A Phylogenomic Approach to Vertebrate Phylogeny Supports a Turtle-Archosaur Affinity and a Possible Paraphyletic Lissamphibia

**DOI:** 10.1371/journal.pone.0048990

**Published:** 2012-11-07

**Authors:** Jonathan J. Fong, Jeremy M. Brown, Matthew K. Fujita, Bastien Boussau

**Affiliations:** 1 Museum of Vertebrate Zoology, University of California, Berkeley, California, United States of America; 2 Department of Integrative Biology, University of California, Berkeley, California, United States of America; 3 College of Natural Sciences, Seoul National University, Seoul, Republic of Korea; 4 Department of Biological Sciences, Louisiana State University, Baton Rouge, Louisiana, United States of America; 5 Museum of Comparative Zoology & Department of Organismic and Evolutionary Biology, Harvard University, Cambridge, Massachusetts, United States of America; 6 Department of Biology, University of Texas-Arlington, Arlington, Texas, United States of America; 7 Laboratorie de Biométrie et Biologie Evolutive, Université de Lyon, Villeurbanne, France; Sars International Centre for Marine Molecular Biology, Norway

## Abstract

In resolving the vertebrate tree of life, two fundamental questions remain: 1) what is the phylogenetic position of turtles within amniotes, and 2) what are the relationships between the three major lissamphibian (extant amphibian) groups? These relationships have historically been difficult to resolve, with five different hypotheses proposed for turtle placement, and four proposed branching patterns within Lissamphibia. We compiled a large cDNA/EST dataset for vertebrates (75 genes for 129 taxa) to address these outstanding questions. Gene-specific phylogenetic analyses revealed a great deal of variation in preferred topology, resulting in topologically ambiguous conclusions from the combined dataset. Due to consistent preferences for the same divergent topologies across genes, we suspected systematic phylogenetic error as a cause of some variation. Accordingly, we developed and tested a novel statistical method that identifies sites that have a high probability of containing biased signal for a specific phylogenetic relationship. After removing putatively biased sites, support emerged for a sister relationship between turtles and either crocodilians or archosaurs, as well as for a caecilian-salamander sister relationship within Lissamphibia, with Lissamphibia potentially paraphyletic.

## Introduction

“The Origin of Species,” and in particular its singular figure, transformed our thinking of biological diversity from the “great chain of being” to the “tree of life” [1]. Resolving the tree of life is crucial to understand organismal evolution and adaptation, but also has far-reaching benefits to diverse fields such as medicine, conservation, and economics [2]. While vertebrates have been the focus of intense phylogenetic research [3]–[5], two fundamental questions in vertebrate systematics remain unanswered: 1) What is the phylogenetic position of turtles within amniotes, and 2) what are the relationships between the three major lissamphibian (extant amphibian) groups–frogs, salamanders, and caecilians?

**Figure 1:**
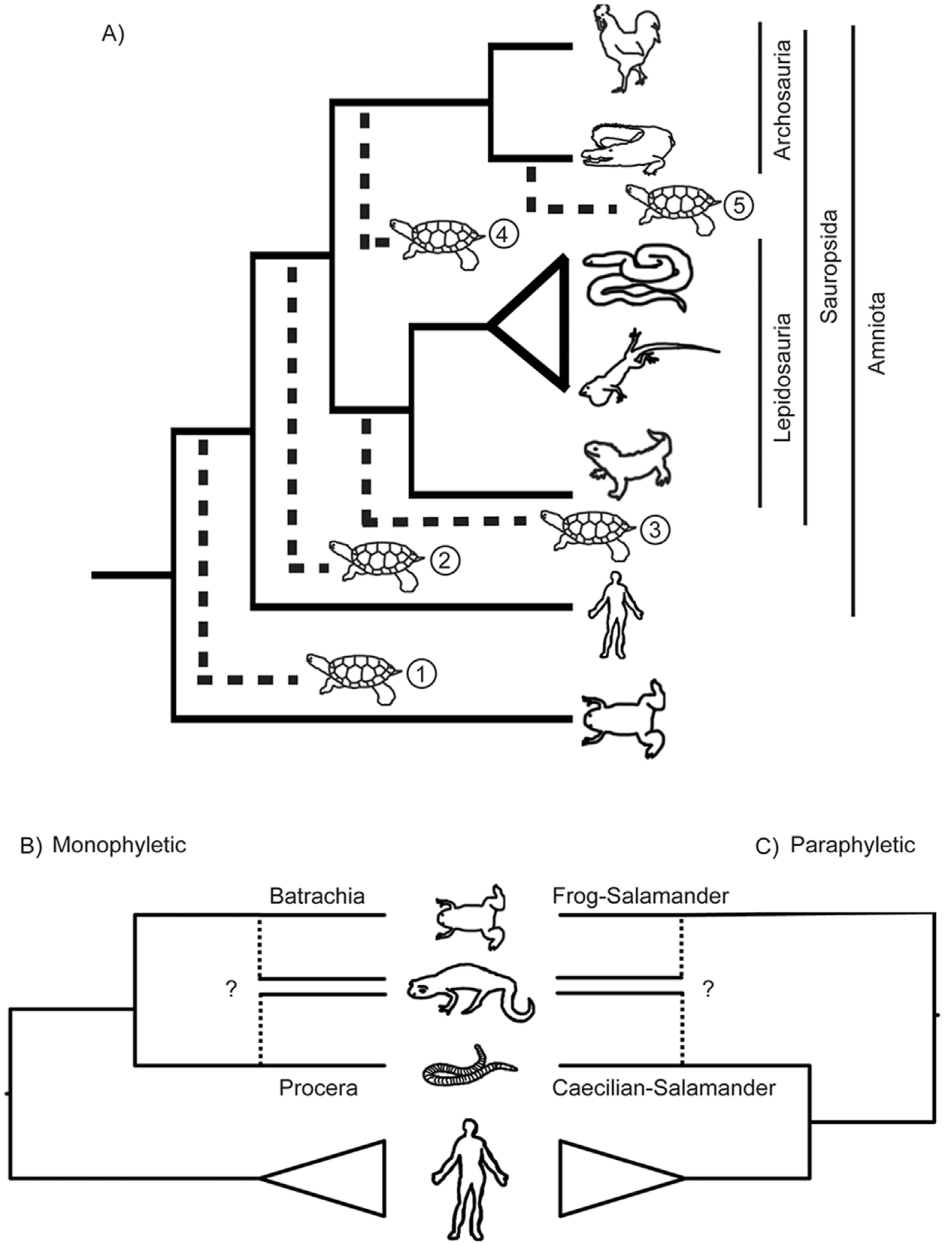
Alternative hypotheses in the vertebrate phylogeny. Uncertainties in the vertebrate phylogeny examined in this study. (A) The five alternative hypotheses for the placement of turtles within amniotes 1) turtles as basal amniotes, 2) turtles as basal sauropsids, 3) turtle-lepidosaur sister group, 4) turtle-archosaur sister group, and 5) turtle-crocodilian sister group. (B) monophyletic and (C) paraphyletic alternative hypotheses for lissamphibian (extant amphibians) relationships.

For more than 150 years, biologists have debated the phylogenetic position of turtles, resulting in no fewer than five different hypotheses (Figure 1A) [4]. Earlier studies used the number of temporal skull openings for classification, with the anapsid condition (no openings) found in turtles, the synapsid condition (single opening) found in mammals, and the diapsid condition (two openings) found in birds and non-turtle reptiles [6]. Morphological and molecular data have suggested four additional hypotheses: turtles as basal sauropsids (reptiles and birds), a turtle-lepidosaur (lizards, snakes, amphisbaenians, and tuatara) sister relationship, a turtle-archosaur (birds and crocodilians) sister relationship, and a turtle-crocodilian sister relationship (Figure 1A) (see [4], [7]–[9] for summary of references). Although recent studies have found strong results supporting specific hypotheses, there is no consensus as different datasets support different hypotheses [7]–[9].

For amphibians, several morphological and physiological characters, including pedicellate teeth and cutaneous respiration, suggest frogs, salamanders, and caecilians share a common origin [10], [11]. However, the monophyly of Lissamphibia is still under debate, as some paleontological studies have inferred a paraphyletic Lissamphibia [12], [13]. There are four proposed branching patterns within Lissamphibia (Figure 1B,C). Two hypotheses, Procera and Batrachia, exhibit a monophyletic Lissamphibia, but differ in the interrelationships among frogs, salamanders, and caecilians. The Procera hypothesis proposes a salamander-caecilian sister relationship (morphology: [14]; mitochondrial DNA: [15], [16]), while the Batrachia hypothesis proposes a frog-salamander sister relationship (morphology: [17]–[19]; nuclear and combined DNA: [20]–[24]) (Figure 1B). Conversely, two hypotheses based primarily on paleontological data suggest that Lissamphibia is paraphyletic because of an affinity between caecilians and amniotes (Figure 1C) [12], [13], with salamanders sister to either frogs [25]–[27] or caecilians [28], [29]. In general, paleontological data support a paraphyletic Lissamphibia, while molecular data support the Batrachia hypothesis.

Both turtles and lissamphibians have ancient divergences within vertebrates (>200 Ma for turtles, frogs, salamanders, and caecilians) [11], [30] and highly modified morphologies. The lack of intermediate forms, either fossil or extant, obscures any obvious morphological evidence of their respective ancestries. Therefore, molecular studies are the best option for uncovering the information necessary to resolve the enigmatic phylogenetic positions of these groups. However, molecular data are not perfect and exhibit several potential pitfalls, especially when trying to resolve difficult phylogenetic questions [31]. For instance, stochastic error (from insufficient data) and/or systematic error (from inadequate models of substitution), can lead to erroneous inferences [32]. Rogue taxa (i.e., taxa with strong support for multiple phylogenetic positions due to either variation across genes or systematic error) can also impede phylogenetic inference by appearing to reduce confidence in other relationships [33].

In this study, we address two difficult phylogenetic questions in the vertebrate phylogeny: the placement of turtles among amniotes and the relationships among frogs, salamanders, and caecilians. Minimizing stochastic error requires acquiring a sizeable dataset suitable for testing the hypotheses of interest. We do this in our study by compiling one of the largest datasets for vertebrate phylogenetics to date (75 genes for 129 taxa). Systematic error is more difficult to address and not solvable by acquiring additional data [31]. Some generalized approaches to address systematic error involve transforming data [34]–[38] or removing fast evolving sites [39] or genes [40] to reduce homoplasy. We test such methods with limited levels of success. We therefore develop a new method to remove those data that are most likely to harbor non-phylogenetic signal. Instead of a generalized removal of fast sites or genes, we take a different approach that identifies and removes sites that have a high probability of containing biased signal for a specific phylogenetic relationship. By minimizing non-phylogenetic signal and removing rogue taxa, ambiguity regarding the preferred hypotheses was greatly reduced, allowing us to infer a sister relationship between turtles and either crocodilians or archosaurs, as well as a sister relationship between caecilians and salamanders. Some support was also found for a paraphyletic Lissamphibia, with the caecilian-salamander clade more closely related to amniotes than to frogs.

## Results

### Dataset Characteristics

We obtained DNA sequences of 75 protein-coding genes for 129 taxa from 1) online genomic resources and 2) targeted sequencing of new samples [41]. Taxon sampling spans all vertebrates, but is skewed towards mammals (36, available data online) and turtles (45, for future studies within turtles). The concatenated alignment of all genes includes 33,938 base positions, and the overall matrix completeness is 41.6% for a total of 4,378,002 bp of sequence data. On average, taxa in the dataset include 31 of the 75 genes. Of the 3,989 sequences, 878 are new (Genbank #s: JF264630-264720, JN864096-864759, JN885182-885183), while the remaining 3,111 are from online resources. In addition to a standard nucleotide dataset [NUCL], we used three transformations of our data to minimize homoplasy for deeper evolutionary divergences: 1) amino acids (AA) [34], [35], 2) first and second codon positions (N12) [36], [37], and 3) sequence adjustment to account for codon degeneracy (DEGEN1) [38]. In addition, for each of these data transformations, we applied four alternative taxonomic and gene sampling strategies: 1) all taxa for all 75 genes (All taxa-75 genes), 2) a subset of 16 taxa (see below) for all 75 genes (16 taxa-75genes), 3) all taxa for a reduced set of genes with sequences from all major taxonomic groups for the turtle (All taxa-31 genes) and lissamphibian (All taxa-26 genes) questions, and 4) 16 taxa for each reduced gene set (Turtle: 16 taxa-31 genes, Lissamphibia: 16 taxa-26 genes). For the 16-taxon datasets, only 2–4 of the most data-complete taxa were included from each major taxonomic group to explore the impacts of missing data, as this dataset is 81.9% complete. Lastly, to investigate the effect of fast evolving genes on phylogenetic reconstruction, we calculated the rate of evolution of each gene and removed the fastest genes from our analyses. Based on the shape of the frequency histogram (Fig. S2), we removed the fastest 25% of genes and concatenated the remaining genes (dataset named ‘slow genes’) for analysis. All data files have been deposited in the Dryad Repository: http://dx.doi.org/10.5061/dryad.25j6h.

### Phylogenetic Analyses and Topology Tests

**Figure 2:**
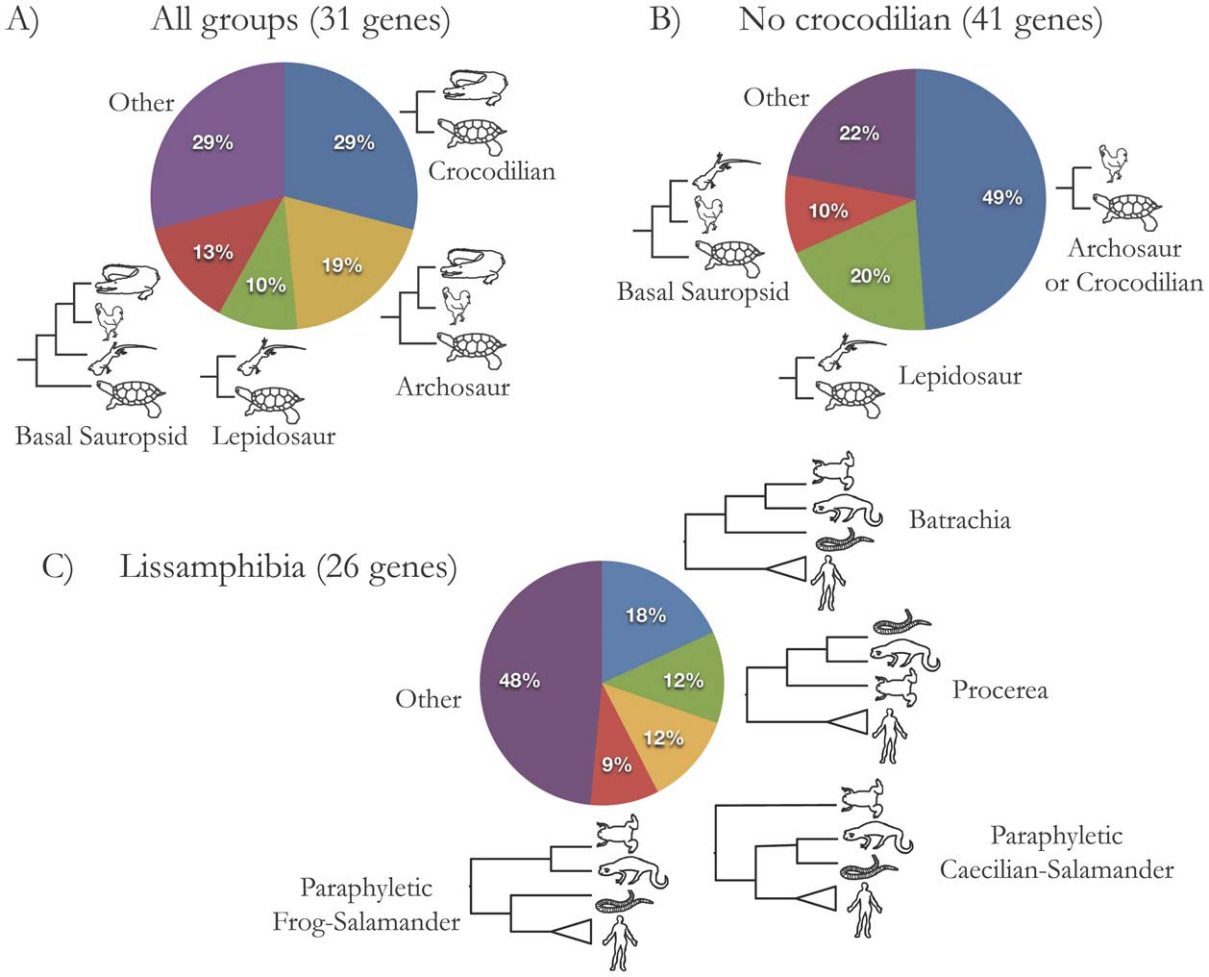
Phylogenetic results from individual gene analyses. (A) The phylogenetic position of turtles within amniotes when all major groups were present and (B) when no crocodilians were present. (C) The relationships between major lissamphibian groups. The “other” category includes topologies that do not match any of the previously proposed hypotheses, usually with a major amniote group being paraphyletic.

We inferred gene trees for each gene using maximum likelihood (RAxML); each hypothesis for both the turtle placement and lissamphibian relationships was supported by subsets of these individual gene trees (Figure 2). For the phylogenetic placement of turtles, when all major amniote groups were included, the turtle-crocodilian topology was most frequently supported (29%), while the turtle-bird topology was most common (49%) when no crocodilian was available (Figure 2A,B). For lissamphibian relationships, the Batrachia hypothesis was most frequently supported by individual gene analyses (18%), but support was more evenly spread across each of the hypotheses compared to the turtle question (Figure 2C). Coalescent-based analyses such as BEST [42] can be used to deal with incongruent gene trees, but were not possible for our datasets, as analyses failed to converge (potentially due to the large amount of missing data [43]). So we focus on the concatenated analyses and their results for the remainder of this paper. Topology tests (approximately unbiased topology tests [AU tests]) [44] were run alongside phylogenetic analyses to rigorously test whether the maximum likelihood tree is statistically better than alternative topologies.

For the phylogenetic placement of turtles, results from concatenated datasets were generally consistent within, but differed between, each data transformation (Figure S1, Table 1). The NUCL data-type recovered a turtle-crocodilian relationship, which was statistically indistinguishable from a turtle-archosaur sister relationship based on AU tests in all datasets (Table 1). For the N12 data-type, a turtle-archosaur relationship was recovered in all but one dataset (16 taxa-31 genes: turtle-lepidosaur), and AU tests statistically excluded turtles as basal amniotes. For the DEGEN1 and AA data-types, the turtle-lepidosaur relationship was recovered for all datasets, but results from AU tests were not consistent.

**Table 1:** Summary of phylogenetic results from different datasets.

		NUCL				N12				DEGEN1				AA				
TURTLE	Individual	All taxa	16 taxa	All taxa	16 taxa	All taxa	16 taxa	All taxa	16 taxa	All taxa	16 taxa	All taxa	16 taxa	All taxa	16 taxa	All taxa	16 taxa	Slow
	Genes	75 genes	75 genes	31 genes	31 genes	75 genes	75 genes	31 genes	31 genes	75 genes	75 genes	31 genes	31 genes	75 genes	75 genes	31 genes	31 genes	Genes
Basal Amniote	0%	0.003	2e-40	3e-7	1e-59	0.001	1e-5	8e-5	0.005	0.001	0.005	**0.274**	**0.237**	0.001	6e-6	**0.477**	**0.32**	5e-6
Basal Sauropsid	13%	3e-4	1e-4	0.001	**0.056**	0.009	**0.188**	**0.32**	**0.383**	**0.07**	**0.351**	**0.424**	**0.454**	**0.117**	**0.137**	**0.398**	**0.586**	5e-4
Turtle-Lepidosaur	10%	2e-5	1e-4	2e-4	0.016	**0.144**	**0.339**	**0.412**	**X**	**X**	**X**	**X**	**X**	**X**	**X**	**X**	**X**	1e-6
Turtle-Archosaur	19%	**0.143**	**0.177**	**0.327**	**0.471**	**X**	**X**	**X**	**0.505**	**0.601**	**0.151**	**0.526**	0.041	**0.345**	**0.295**	**0.283**	**0.027**	**0.477**
Turtle-Crocodilian	29%	**X**	**X**	**X**	**X**	**0.428**	**0.489**	**0.448**	**0.31**	**0.094**	**0.062**	**0.152**	0.007	**0.094**	0.038	**0.167**	0.006	**X**
Other	29%	–	–	–	–	–	–	–	–	–	–	–	–	–	–	–	–	–
		**NUCL**				**N12**				**DEGEN1**				**AA**				
**LISSAMPHIBIA**	**Individual**	**All taxa**	**16 taxa**	**All taxa**	**16 taxa**	**All taxa**	**16 taxa**	**All taxa**	**16 taxa**	**All taxa**	**16 taxa**	**All taxa**	**16 taxa**	**All taxa**	**16 taxa**	**All taxa**	**16 taxa**	**Slow**
	**Genes**	**75 genes**	**75 genes**	**26 genes**	**26 genes**	**75 genes**	**75 genes**	**26 genes**	**26 genes**	**75 genes**	**75 genes**	**26 genes**	**26 genes**	**75 genes**	**75 genes**	**26 genes**	**26 genes**	**Genes**
Batrachia	18%	0.009	0.008	**0.204**	**0.402**	**X**	**X**	**X**	**X**	**X**	**0.643**	**X**	**X**	**0.539**	**X**	**X**	**0.644**	**0.234**
Procera	12%	0.002	0.003	0.037	**0.069**	**0.311**	**0.339**	**0.29**	**0.183**	**0.394**	**X**	**0.419**	**0.215**	**0.334**	**0.343**	**0.169**	**0.094**	**0.087**
Paraphyletic																		
Frog-Salamander	9%	0.001	0.001	0.004	**0.153**	**0.512**	**0.587**	**0.273**	**0.403**	**0.386**	**0.087**	**0.269**	**0.074**	**X**	**0.337**	**0.361**	**0.235**	**0.188**
Paraphyletic																		
Caecilian-Salamander	12%	**0.103**	**0.209**	**0.184**	**0.093**	**0.185**	**0.198**	**0.061**	**0.013**	**0.166**	**0.093**	**0.074**	**0.012**	**0.23**	**0.224**	0.042	**0.018**	**0.873**
Other	48%	**X**	**X**	**X**	**X**	–	–	–	–	–	–	–	–	–	–	–	**X**	**X**

Summary of results from phylogenetic analyses for turtle placement within the amniote phylogeny, and relationships of Lissamphibia (extant amphibians). Individual gene and concatenated analyses were performed. For individual gene analyses, percentages denote the proportion of genes supporting the hypothesis. For the concatenated analyses, the cell with “X” for each column denote the most likely topology based on RAxML likelihood scores, while the numbers in cells represent p-values based on approximately unbiased topology tests (Shimodaira 2002). Cells in bold font denote statistically indistinguishable topologies (p-value>0.05) from the most likely topology. Sixteen total concatenated analyses were performed (4 data transformations × 4 datasets). Data were transformed in an attempt to reduce the rate of evolution: NUCL = complete nucleotide dataset, N12 = 1^st^ and 2^nd^ codon positions only (Edwards et al. 1991, Blouin et al. 1998), DEGEN1 = codon degeneracy (Regier et al. 2010), AA = amino acids (Meyer 1994). Three different datasets were compiled in attempts to minimize the amount of missing data: 16 taxa (reduced taxon set to include the taxa with the most complete data for each major vertebrate group), 31 genes (for turtle question, genes with representatives from all the major groups in question), 26 genes (for lissamphibian question, genes with representatives from all the major groups in question). The last column summarizes the results from phylogenetic analyses when removing the 19 most quickly evolving genes (25% of total genes).

For the lissamphibian question, phylogenetic analyses for N12, DEGEN1, and AA often recovered different relationships, but most AU tests did not exclude any of the four major hypotheses. The NUCL data-type was unique in that the recovered topology had no two lissamphibian groups monophyletic (Figure S1), but of the four major hypotheses, provided the most support for a paraphyletic Lissamphibia, with a caecilian-salamander clade (Table 1).

Results from phylogenetic analyses and AU tests performed on the ‘slow genes’ dataset are summarized in Table 1. For the turtle question, a turtle-crocodile relationship was recovered, which was statistically indistinguishable from a turtle-archosaur relationship. For the lissamphibian question, results were identical to the NUCL data-type where no two lissamphibian groups were monophyletic, and AU tests could not statistically reject any of the four hypotheses.

### Rogue Taxa Analyses

Unstable (rogue) taxa in a phylogeny can affect phylogenetic inference. Removal of these taxa can improve phylogenetic results by increasing resolution and/or support values [33]. We identified 19–39 rogue taxa for each of the four data-types, with much overlap between data-types. Although phylogenetic relationships of major groups were the same, removal of rogue taxa improved analyses by increasing bootstrap support values of clades.

### Statistical Analyses

**Figure 3:**
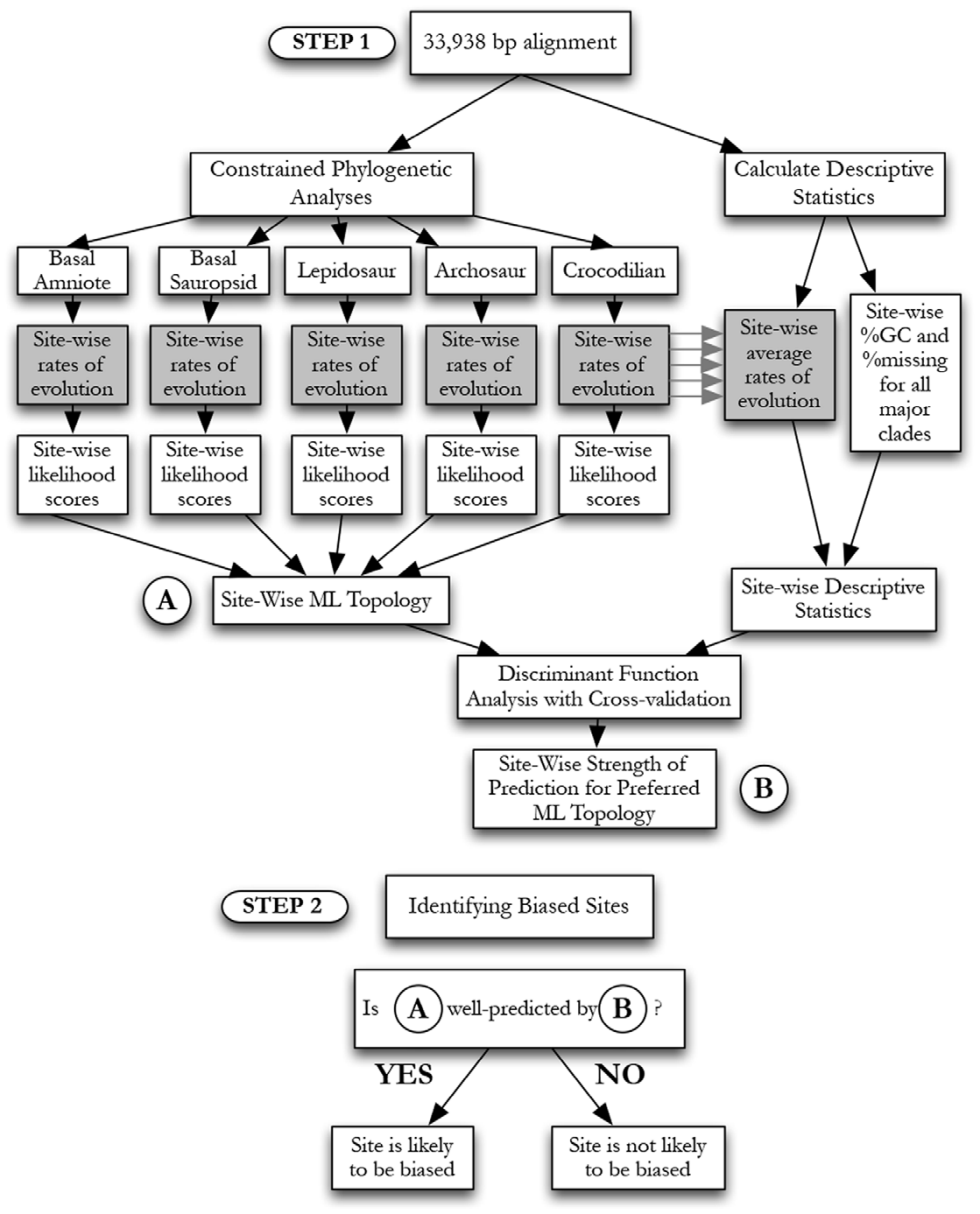
Flow diagram of data filtering method. Steps of the new statistical methodology to identify and filter out sites that contain putative non-phylogenetic signal (i.e. biased sites). Analyses pertaining to the phylogenetic position of turtles are used in this example.

Initial phylogenetic results were inconclusive, possibly due to conflicts between phylogenetic and non-phylogenetic signal. Features of the data that may be correlated with biases in phylogenetic reconstruction include site-specific rates of evolution (site-rates), as well as heterogeneities between clades in GC content (%GC) and amount of missing data (%missing) [45], [46]. We reason that if these correlates of non-phylogenetic signal alone can do a good job of predicting the phylogeny favored by a site in the alignment, this site is likely to be biased and cannot be trusted. A diagram of our methodology to identify biased sites can be found in Figure 3. First, we compute site-rates for each site in the alignment, and %GC and %missing per site for major clades relevant to turtle placement and lissamphibian relationships. In addition, we compute site-wise likelihoods for all competing hypotheses regarding the phylogenetic positions of turtles and Lissamphibia and recorded the topology with the highest likelihood for each site. Next, we use Discriminant Function Analysis (DFA; employing a quadratic discriminant function) to predict the favored topology based solely on descriptive statistics (site-rates, %GC, and %missing). Based on the strength with which the DFA was able to predict the topology preferred by any site, we designated sites as putatively biased and progressively removed them from the analysis.

We validated our approach on simulated data and a previously published biological dataset [47]. We simulated sequences along a tree with 8 leaves under strong heterogeneities in rates of evolution among sites, relative branch lengths among sites, and equilibrium %GC among taxa (see methods). Phylogenetic reconstruction using all sites without filtering resulted in an artifactual topology where species with similar %GC and high rates of evolution clustered together. We computed %GC and site-rates (simulated dataset was complete, without missing data) on the simulated sequences, and used our procedure to filter sites using site-rates only or both site-rates and %GC. Although the sequences had been simulated under strong compositional heterogeneity, filtering based on site-rates only resulted in better overall results. In fact, the removal of putatively biased sites resulted in the recovery of the correct topology at all thresholds tested. In contrast, filtering based on both %GC and site-rates resulted in the recovery of the correct topology only when removing the largest proportion of sites (Table S1). Contrary to site-rates, %GC contains a complex mixture of phylogenetic and biased signal, which may confuse the method, as shown by the following toy example. If one considers 3 clades A, B, C, with the correct topology ((A,B),C) and convergence towards higher GC content in clades B and C leads to the artifactual topology (A,(B,C)). High GC contents in clade B, clade C, or even in both clades B and C are not by themselves sufficient for predicting that a site is likely to provide biased signal. Only in the case where A is GC poor and both B and C are GC rich can this site be safely assumed to likely provide biased signal. All seven other configurations (all three clades GC rich; the two other configurations with two clades GC rich, the three configurations where two clades are GC poor, and three clades GC poor) are not indicative of a compositional artifact. Consequently, to predict putatively biased sites using compositional statistics for clades, a complex interaction between three variables has to be uncovered by the method. As our DFA does not consider interaction terms between two or more variables, it cannot perform well with %GC. Other predictor variables (e.g., site-rates or %missing) may not require interactions between two or more variables for predicting putatively biased sites, and are thus more amenable to our analysis through DFA. For instance, the rate of a site or the percent of missing data in a particular clade could be enough to predict that a site has the potential for providing biased signal.

To further validate our approach, we used a dataset of eight gene concatenates addressing the Ecdysozoa-Coelomata controversy [48]. In their paper, Wolf et al. (2003) [47] concluded in favor of the Coelomata hypothesis, as analyses of the datasets resulted in 5/8 topologies strongly supporting Coelomata. However, most recent studies support the Ecdysozoa hypothesis and suggest that the Coelomata hypothesis is an artifactual result linked to fast-evolving taxa and inadequate taxonomic sampling [49]. The original dataset was a complete, amino acid dataset, so we are unable to calculate %GC and %missing. Therefore, we only computed site-rates and applied our filtering procedure on the eight datasets, comparing it to random removal of sites as a control. After filtering, 6/8 alignments support the Ecdysozoa hypothesis (Table S2), changing the support of three genes from Coelomata to Ecdysozoa. These results suggest that our approach had successfully filtered out biased signal from the alignments.

We computed site-wise descriptive statistics and most likely topologies for the NUCL dataset. As our method focuses on specific phylogenetic questions, we performed filtering of biased sites twice, once for the turtle question and once for the Lissamphibia question, producing two different sets of alignments.

We find that DFA accurately predicts the most likely topology for 47% of the sites for Lissamphibia, and 36% of the sites for turtles. DFA is able to predict the topology with the highest site likelihood more accurately than the control (see methods; sites are correctly predicted by the DFA analysis 1.55× and 1.65× more often than random expectations for Lissamphibia and turtles, respectively) (Table S3). The predictive ability of DFA is significantly better than expected at random, based on the results of permutation tests (Figure S3).

Interestingly, the ability of DFA to predict the preferred topology at a site varies by topology. In lissamphibians, DFA is most able to predict the Procera topology (1.98× more accurately than the control predictor) and least able to predict the Batrachia topology (1.43×). In turtles, DFA is most able to predict the Lepidosaur topology (3.62×) and least able to predict the Archosaur topology (0.66×) (Table S3). For each site, DFA can also be used to calculate a support value corresponding to the strength of its prediction. For instance, regarding Lissamphibia, the 1% most confidently predicted sites based on DFA all support the Procera hypothesis, and for turtles, the 1% most confidently predicted sites all support the Sauropsid topology. This shows that the Procera topology for lissamphibian relationships, and the Lepidosaur and Sauropsid topologies for turtle placement can be predicted by characteristics of the sites that should be unrelated to the site’s preferred topology, and suggests they may be supported in part by non-phylogenetic signal in the alignment. We note that all four candidate topologies for Lissamphibia are predicted with similar accuracies by the DFA analysis, in contrast with the turtle analysis. This may imply that the biased signal we detect is more equally distributed among the different lissamphibian hypotheses than for the turtle hypotheses.

Based on the performance of DFA-filtering when analyzing simulated as well as empirical data, we performed two DFA analyses: three types of descriptive statistics (site-rates, %GC, and %missing) or two types (excluding %GC). We generated several alignments by removing the 10%, 20%, 30%, 40%, or 50% most confidently predicted (i.e. most suspect) sites from the alignment for the turtle and Lissamphibia analyses, and generated phylogenies from these sub-sampled alignments as well as alignments of the discarded sites. For turtles, all phylogenetic analyses and topology tests based on DFA-filtering using all three descriptive statistics support turtles as the sister group to crocodilians (Table S4). Filtered datasets generated without the use of clade-specific %GC as a predictor supported either turtle-crocodilian or turtle-archosaur relationships (Table S4). For Lissamphibia, all analyses using all three descriptive statistics support the same topology in which Lissamphibians are paraphyletic and a caecilian-salamander clade forms the sister group to amniotes (Table S4). However, for analyses excluding %GC, two hypotheses (Procera and Paraphyletic Caecilian-Salamander) are often statistically indistinguishable. Additionally, when excluding %GC and removing 50% and 40% of the data, supported topologies do not match any of the four proposed hypotheses (Table S4). The low bootstrap support values suggest these highly unlikely topologies come from an absence of a clear phylogenetic signal in the remaining sites.

**Figure 4:**
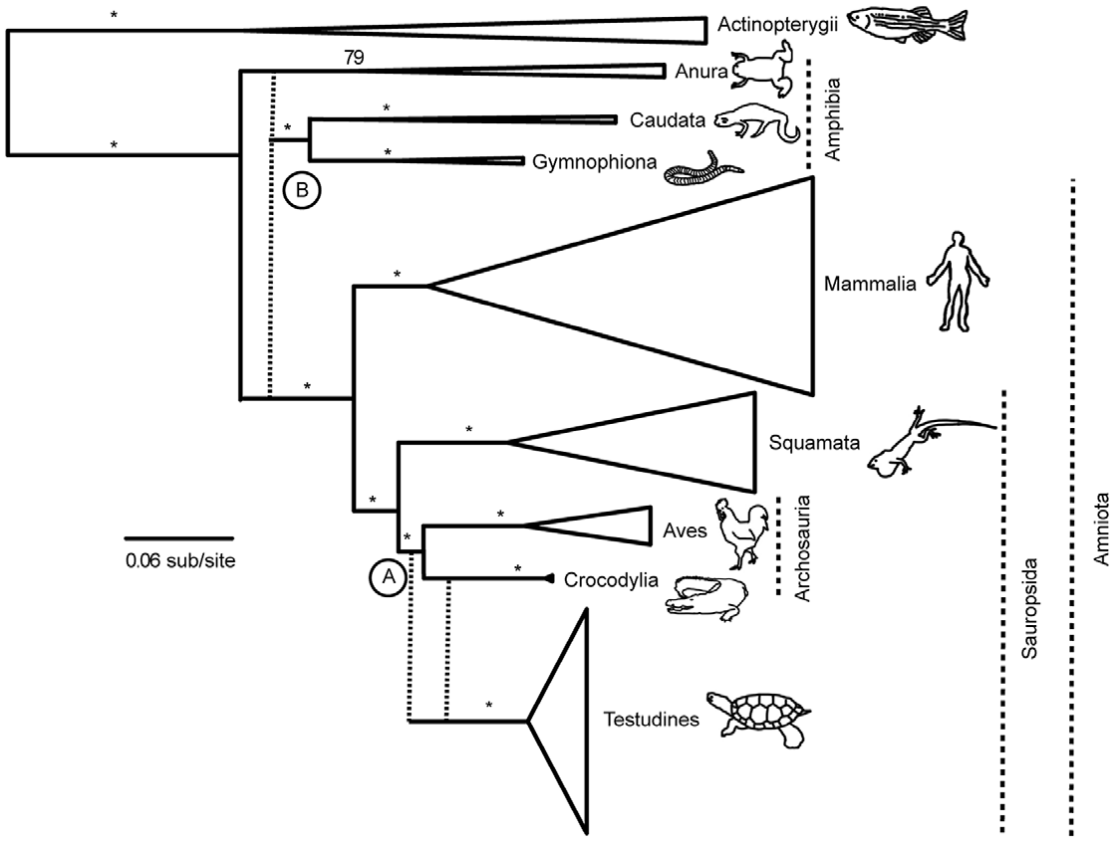
Consensus vertebrate phylogeny. Consensus phylogeny from datasets with the 10% most putatively biased sites removed. (A) Turtles are either the sister group to Crocodilians or Archosauria. (B) Lissamphibia: salamanders (Caudata) and caecilians (Gymnophiona) are sister groups, and this group is either the sister group to frogs (Procera hypothesis) or Amniota (rendering Lissamphibia paraphyletic). RAxML bootstrap values are at nodes, with “*” representing support ≥95.

From the four alignments with the 10% most suspect data removed, one for each combination of taxonomic question and number of DFA predictor types (2 or 3), we can exclude all but four possible topologies relating major vertebrate groups. We combine these trees to produce a consensus phylogeny, with relationships within amniotes from the turtle datasets and deeper vertebrate relationships from the lissamphibian datasets. The consensus phylogeny of higher-level vertebrate relationships from our study is in Figure 4.

## Discussion

Previous studies of the vertebrate phylogeny have resulted in ambiguity regarding the phylogenetic placement of turtles within amniotes and the interrelationships within Lissamphibia (Figure 1), in part because the short internodes and long branches that characterize these groups are notoriously difficult problems in phylogenetic inference. Using standard phylogenetic approaches, past studies – as well as similar efforts with our data – have not yielded consistent results (see [5], [12]). We believe that difficult phylogenetic problems, such as these, could be due to the presence of conflicting phylogenetic signal in the dataset. In large datasets, the problem may not be the amount of phylogenetic signal, but rather the confounding effects of phylogenetic error. Philippe et al. (2011) [31] outline three primary sources of phylogenetic error: 1) incorrect identification of orthologs, 2) erroneous sequence alignments, and 3) inadequate models of evolution. The first two points are addressed in our dataset by rigorously testing orthology and alignment through the marker development and data analysis stages [41]. Some standard methods to address the third point are to reduce homoplasy by transforming data and removing genes. For our study, data transformations were ineffective at removing conflicting signal, while removal of fast evolving genes was partially successful, but conflicting signal remained, especially for the lissamphibian question. Accordingly we developed a new method that predicts and removes potentially biased sites for a specific phylogenetic question. Our method tests the potential for biased inferences to result from a high rate of evolution as well as two other potential contributors of non-phylogenetic signal [45], [46]: GC content (%GC) and proportion of missing data (%missing). Our simulations and tests on empirical data showed that our approach is promising in its ability to remove biased signal, notably when %GC is not included as a predictive variable. When we implemented this statistical procedure to filter our data, we reduced conflicting signal and recovered stronger support for higher-level vertebrate relationships.

### Phylogenetic Position of Turtles

Past studies have hypothesized five different phylogenetic positions for turtles in the amniote phylogeny, with the most recent molecular studies debating between the turtle-lepidosaur [7] and turtle-archosaur [8], [9] relationships. Removing the set of sites identified by DFA to have the greatest chance of contributing biased signal allowed statistical exclusion of three previously proposed hypotheses: a turtle-lepidosauria sister grouping, turtles as basal sauropids (reptiles and birds), and turtles as basal amniotes (Table 2). Our results show that turtles are closely related to birds and crocodilians, but since results differed when clade-specific %GC content was or was not included in the set of predictor variables (Table 2), we are not able to distinguish between the turtle-archosaur and turtle-crocodilian topologies.

**Table 2:** Phylogenetic results from filtered datasets.

	3 types (site-rates, %GC, %missing)					2 types (site-rates, %missing)				
TURTLE	50%	40%	30%	20%	10%	50%	40%	30%	20%	10%
Archosaur	7e-5	2e-5	1e-4	1e-4	0.031	**X**	**X**	**X**	**0.313**	**0.173**
Crocodilian	**X**	**X**	**X**	**X**	**X**	0.006	**0.119**	**0.32**	**X**	**X**
Lepidosaur	6e-79	5e-33	8e-98	2e-54	6e-6	1e-68	1e-7	2e-59	1e-46	1e-48
Basal Sauropsid	4e-5	3e-117	4e-5	2e-8	2e-4	8e-65	2e-50	2e-70	1e-56	1e-44
Basal Amniote	6e-5	7e-10	1e-6	1e-84	0.001	5e-26	1e-4	5e-7	1e-6	12e-5
	**3 types (site-rates, %GC, %missing)**					**2 types (site-rates, %missing)**				
**LISSAMPHIBIA**	**50%**	**40%**	**30%**	**20%**	**10%**	**50%**	**40%**	**30%**	**20%**	**10%**
Batrachia	7e-36	7e-34	3e-6	2e-65	4e-52	0.004	0.003	0.019	0.015	0.003
Procera	7e-11	5e-61	5e-5	2e-66	93–94	0.029	**0.405**	**0.375**	**0.131**	3e-4
Paraphyletic										
Frog-Salamander	3e-76	2e-7	1e-6	6e-67	5e-6	2e-7	0.004	1e-4	0.002	0.001
Paraphyletic										
Caecilian-Salamander	**X**	**X**	**X**	**X**	**X**	7e-11	0.036	**X**	**X**	**X**
Other	–	–	–	–	–	**X**	**X**	–	–	–

Varying amounts of suspect sites were removed and tested. A) Position of turtles in the amniote phylogeny using three descriptive statistics (site-rates, %GC, and %missing), B) position of turtles in the amniote phylogeny using two descriptive statistics (excluding %GC), C) interrelationships of Lissamphibian groups using three descriptive statistics (site-rates, %GC, and %missing), D) interrelationships of Lissamphibian groups using two descriptive statistics (excluding %GC). The percentage in each column represents the percentage of sites removed from the dataset. Values in cells represent p-values, “X” denotes the best tree, and trees statistically indistinguishable from the best tree are in bold font (Approximately Unbiased topology test p-value >5%).

Recent results [8], [9], as well as our findings, placing turtles as close relatives of crocodilians and birds, necessitates changing the traditional view of turtle evolution, as it prevailed until recently. First, Archosauria is defined as the crown group including the most recent common ancestor of birds and crocodilians [50]; turtles are either the sister group to or a member of Archosauria. A recent paleontological study supports this relationship between archosaurs and turtles, discovering a unique skull ossification (laterosphenoid) found only in turtles and Archosauriformes [51].

An important question in turtle biology is how and when its unique, shelled body plan evolved. Previous work suggested parareptilian (Anapsida) groups as the extinct ancestor of turtles [52], [53], with one hypothesis pointing towards the elaboration of dermal armor as a precursor to formation of the shell [54]. With our results nesting turtles among diapsids, hypotheses of turtle shell evolution from parareptilian ancestors are no longer possible. Turtles are unique in that their ribs develop by encapsulating the shoulder blades and embed within the dermis, sending developmental signals to the dermis to form bone and therefore the carapace [55]. Understanding how and when the turtle shell arose will come only from studying extinct archosaurian lineages.

### Relationships within Lissamphibia

Ancestral amphibians appear in the fossil record starting in the late Devonian and are extremely diverse in the Palaeozoic. However, a large gap in the fossil record exists between Palaeozoic amphibians and lissamphibians, with the exception of Stereospondyls extending into the Mesozoic [56] and a possible frog ancestor found in the Lower Triassic [12]. It is this gap in the fossil record paired with significant morphological change that has made it difficult to determine the ancestors of and relationships among modern amphibians from paleontological data.

The most recent molecular study based on mitochondrial genomes and eight nuclear genes [24] supports the Batrachia hypothesis, which is in contrast to most paleontological studies supporting a paraphyletic Lissamphibia [12], [13], [25]–[29]. This raises the question of whether morphological or molecular data are correct [26]. The results of our molecular study supports a caecilian-salamander sister relationship, but cannot distinguish between a monophyletic and paraphyletic Lissamphibia (Figure 4). Although our results do not resolve the lissamphibian origin problem, we resurrect an often-overlooked hypothesis of a paraphyletic Lissamphibia. This is the first molecular study to have signal supporting a paraphyletic Lissamphibia, allowing for the possibility of concordance between morphological and molecular data. If the paraphyletic hypothesis is true, caecilians and salamanders would be more closely related to humans and other amniotes than to frogs. To discriminate between the alternative hypotheses of a monophyletic and paraphyletic Lissamphibia, deeper taxon and gene sampling for lissamphibian groups will probably be needed.

### Removal of Biased Signal

A common notion in molecular systematics is that the solution to resolving difficult relationships is to include ever increasing amounts of data. This belief is based on the idea that true phylogenetic signal will eventually dominate and drive the results of an analysis, circumventing any methodological problems. However, our results suggest that inclusion of more data can introduce biased signal into a dataset, resulting in a lack of resolution or even misleading inferences, a possibility also raised by others [31]. Methods to filter data based on the rate of evolution have been previously used to increase resolution by removing non-phylogenetic signal [39], [40]. In an effort to remove non-phylogenetic signal in a more targeted way, we develop a new DFA-based filtering method that attempts to identify sites contributing biased signal based on several data set characteristics known to cause inference problems in certain contexts (site-rates, %GC, %missing). In addition to using more information for predicting non-phylogenetic signal, our approach is different from other methods because it targets a specific phylogenetic relationship (i.e. sites identified as biased for the turtle question are specific to that question). We anticipate and encourage future studies that will more fully assess which characteristics of sites can be used with this method to accurately predict the presence of phylogenetic bias (e.g. whether or not %GC can improve the prediction of bias and, if so, when). Future studies could also assess potential performance gains from including interaction terms among variables in the DFA or including information about the strength with which a particular site supports its preferred phylogenetic hypothesis.

Proper modeling of molecular evolution and evaluation of the fit between data and model seem to be just as important as the amount of data present in a study. With the advent of new technologies that produce sequence data faster and more cheaply than ever before, datasets will only become larger, and issues relating to signal quality will become even more important in molecular systematics. We view our DFA approach as an important step towards the goal of objectively identifying non-phylogenetic signal in large datasets.

### Conclusion

With increasingly large datasets being gathered for phylogenetics, many relationships have been confidently resolved. What remains are controversial, difficult to resolve phylogenetic questions, probably arising from conflicting and biased phylogenetic signal in the data. We developed a method for identifying and minimizing biases from molecular data to tackle two persistent yet fundamental problems in vertebrate phylogenetics: the placement of turtles within amniotes and the interrelationships within Lissamphibia. Based on tests of our filtering method on simulated and empirical datasets, we believe that we are able to reduce the amount of conflicting signal in datasets. For the vertebrate phylogeny, the application of this filtering method results in analyses that support turtles being closely related to archosaurs, as either the sister group to crocodilians or archosaurs, and a caecilian-salamander sister relationship, with the possible paraphyly of Lissamphibia. Because of our use of a new statistical approach, we view our results to be tentative and encourage more work from paleontologists and molecular biologists alike to further evaluate these hypotheses and methodology. Given the importance of the historical framework provided by phylogenetic systematics in fields as diverse as developmental biology, genomics, conservation biology and paleontology, we believe approaches like ours will be useful to resolve major phylogenetic questions and advance modern biological thought.

## Materials and Methods

### Ethics Statement

This research was conducted under and approved by UC Berkeley's Animal Care and Use Committee (protocol #R279-0211). Tissue samples used in this study were obtained from the Museum of Vertebrate Zoology (MVZ), an institution that serves as a specimen and tissue repository for researchers. The MVZ has a strict policy for researchers when depositing specimens and tissues into the museum, requiring local collecting permits and import permits when necessary.

### Taxon Sampling

Our study included sampling for all major vertebrate groups except Tuatara (*Sphenodon*). Omission of the Tuatara is inconsequential to our investigation due to its uncontroversial affinity with Squamata ( = Lepidosauria) [57]. Of the 129 taxa included in this study, data were available for 46 taxa from complete genomes or ESTs (GenBank and Ensembl [58]), and the remaining 82 samples were newly sequenced. Available data were skewed heavily towards mammals (36 taxa), but also include fish (5 taxa), frog (1 taxon), salamander (1 taxon), lizard (1 taxon), bird (2 taxa), and crocodile (1 taxon). New taxon sampling expanded representation within amphibians, reptiles, and birds. A detailed list of all taxa used in this study, along with Genbank numbers, can be found in Table S5.

### Marker Sampling

Markers used in this study are single-copy, orthologous, protein-coding genes [41]. The single-copy nature of the markers was checked both during marker development and after data collection. During marker development, markers were pre-screened using BLAST to compare with the high-coverage, well-annotated chicken genome and gene families were identified in Ensembl and Metazome. After data collection, to identify and remove paralogous genes, preliminary phylogenetic analyses using RAxML [59] were performed for each gene. Each of the trees was analyzed individually by eye for erroneous phylogenetic relationships (e.g. mammal species more closely related to fish) and signatures of gene duplications (i.e. replicated tree topologies within the larger tree). Sequences with erroneous positions were removed, and when gene duplications were detected, the sub-tree that included newly sequenced data was retained for subsequent analyses. We obtained new sequences according to the methods of Fong & Fujita (2011) [41]. Briefly, we used cDNA preparations as template to amplify the protein-coding genes using conserved primers. Amplicons were sequenced using ABI3730 chemistry, and sequences were edited using Geneious 5 (Biomatters Ltd.) and aligned using MUSCLE [60].

### Datasets and Data-types

Sequences were combined into two main categories of datasets: individual genes and concatenations. Individual datasets for the 75 genes consisted of orthologous sequences from online genomes and the new samples. Combining individual genes using a Perl script (available upon request) produced the concatenated datasets. We compiled seven different concatenated datasets: 1) All taxa-75 genes, 2) 16 taxa-75 genes, 3) All taxa-31 genes (turtle), 4) All taxa-26 genes (Lissamphibia), 5) 16 taxa-31 genes (turtle), 6) 16 taxa-26 genes (Lissamphibia), 7) slow genes (removal of fastest 25% of genes). For the 16-taxon datasets, the vertebrate group Crocodilia is represented by two individuals of the species *Alligator mississippiensis* (from the EST database and a new sample). We combined the data from both individuals to minimize missing data; this approach is justified, as when there were data from both individuals for a marker, data were identical. The dataset of reduced loci for all taxa was used when evaluating the specific phylogenetic questions (turtle and Lissamphibia). Loci without representatives of all the focal groups were removed, leaving 31 genes for the turtle analysis and 26 genes for the lissamphibian analysis.

The standard nucleotide (NUCL) dataset was transformed to three data-types using the following methods. AA was translated in Geneious 5 (Biomatters Ltd.), 3^rd^ codon positions were removed for N12 using MacClade v4.08 [61], and DEGEN1 was converted using a Perl script [38].

The rate of evolution for each of the 75 genes was calculated by computing tree length and averaging branch lengths using an R script [62]. Based on the shape of the frequency histogram (Fig. S2), we drew a cut-off of average branch length of 0.04, which denoted the top 25% fastest genes (19 genes) for removal. The names of these genes are: DSCR3, EXOC2, GAPDH, GDE1, GNAS, HPD, H2AFY2, IFT57, MAT2B, OAT, OAZ1, PPM1A, PPTC7, PSAT1, SEC13, SGK1, TAT, UBE2J2, and XPOT (Table S5) [41].

### Phylogenetic Analyses

All datasets were subject to maximum likelihood analyses using RAxML [59], and a subset of datasets were also subject to Bayesian analyses using MrBayes [63].

Since this study deals with a complex, multi-gene dataset, we explored heterogeneous processes of molecular evolution through partitioning the data. Tests of alternative partitioning strategies were performed on the NUCL dataset only, as the N12 dataset is a subset of the NUCL dataset, and the DEGEN1 and AA datasets have information on codon position integrated into gene partitions. For RAxML analyses of the NUCL dataset, three different partitioning strategies were tested: by gene (75 partitions), by gene and 1^st^+2^nd^ and 3^rd^ codon position (150 partitions), and by gene and codon position (225 partitions). Likelihood ratio tests selected the 150 partitions as the best partitioning strategy.

RAxML nucleotide analyses used the GTRGAMMA model of evolution for tree inference and bootstrapping (1,000 replicates). RAxML amino acid analyses employed the protein gamma model of evolution and the appropriate model of protein evolution selected using ProtTest v2.4 [64], with empirical amino-acid frequencies for both tree inference and bootstrapping (1,000 replicates). All concatenated datasets were partitioned according to the optimal partitioning strategy. RAxML v7.2.5 and v7.2.6 [59], [65] analyses were run locally and on the CIPRES portal v2.2 [66].

For individual gene analyses, the support values of clades are generally very low, since these relatively short genes (average length is ∼450bp) were used to infer the entire vertebrate phylogeny. However, to understand and summarize the phylogenetic signal for each gene, we classified them based on preferred topology (see Figure 2) irrespective of nodal support.

Bayesian analyses were only run on individual genes and 16-taxon datasets, as the computational burden for the larger datasets would require extremely long analysis times to achieve stationarity (i.e. >2000 hours). When both RAxML and MrBayes analyses were run, preferred topologies were almost identical, so results should not be compromised by reporting only inferences from RAxML. MrBayes v3.1.2 and v3.2 [63] analyses were run locally and using the BioHPC@CBSU resource at Cornell University (http://cbsuapps.tc.cornell.edu). All analyses were run with four chains for 10 million generations. Appropriate models of DNA substitution for each partition were selected using MrModeltest v2.3 [67], and amino acid substitution models were the same as those used in RAxML analyses. Burn-in of MCMC chains was evaluated using the online program AWTY, examining cumulative plots of posterior probabilities of the 20 most variable splits [68].

### Rogue Taxa Analyses

Rogue taxa analyses were performed using RAxML (Stamatakis 2006) and an algorithm described in Pattengale et al. (2010) [33]. Determining the set of rogue taxa to remove was a multi-step process that was run on the concatenated datasets, separately for each of the four data-types. First, a preliminary RAxML analysis was run with all 129 taxa. Based on this phylogeny, taxa that were obviously in incorrect phylogenetic positions (e.g. turtle placed in the mammal clade) were manually removed. These removed taxa tended to be those with the highest levels of missing data. This was repeated until all remaining taxa were placed in the correct clades. Next, the bootstrap results from the RAxML analysis were used as the input data in rogue taxa analyses. The rogue taxa analysis was run 20 times in total, 10 each for both the strict and majority rules consensus trees, using the “−r” randomization option to select a dropset. To maximize the number of taxa retained and remove the most unstable taxa, taxa were considered rogues if they were identified in ≥5 of the 10 analyses under either strict or majority rules consensus. These taxa were removed and steps repeated until there were no rogue taxa identified or no improvement in bootstrap values.

### Topology Tests

Approximately unbiased topology tests (AU tests) [44] were used to test whether sub-optimal trees were significantly worse than the maximum likelihood tree. AU tests were performed to compare the five turtle and four lissamphibian alternative hypotheses for each of the different datasets. Constrained RAxML analyses were run for each of the different topologies using the GTRGAMMA model of sequence evolution, and per-site log likelihoods calculated. These per-site log likelihoods were then input into the program CONSEL [69].

### Discriminant Function Analyses (DFA)

Scripts for DFA analyses of the NUCL dataset were written using the R language [62] and rely on the SeqinR [70] and MASS [71] libraries. These scripts are available from the authors upon request. Per-site log-likelihood scores (LLS) were calculated for each of the constrained phylogenies pertaining to a relevant hypothesis (five turtle positions, four Lissamphibia hypotheses) using RAxML. Site-wise GC content (%GC) and proportion of missing data (%missing) were computed for major clades with potential sister-relationships for turtle placement (turtles, archosaurs, crocodilians, reptiles excluding turtles, and amniotes excluding turtles) and lissamphibian relationships (amniotes, caecilians, caecilians+salamanders, frogs+salamanders, and lissamphibians). Site-specific rates of evolution (site-rates) were calculated for each of the nine constrained phylogenies using HyPhy [72] under a GTR model of sequence evolution with model parameters estimated independently for each phylogenetic hypothesis. These rates were then averaged across the five turtle and four lissamphibian topologies. %GC and %missing were both calculated for each site in the NUCL data matrix, averaged across all taxa in each clade of interest.

DFA (from the MASS library) was run with preferred topology as the predicted category, and %GC (for relevant clades), %missing (for relevant clades), and site-rates as predictor variables in one case, and without %GC in another case. Posterior probabilities from the DFA were calculated using leave-one-out cross-validation and normalized with prior probabilities (posterior/prior ratio). The prior probability of assignment to any particular topology was simply the proportion of sites in the alignment preferring that topology. Two different types of DFA were tested to maximize the predictive power of our analysis: linear discriminant analysis (LDA), and quadratic discriminant analysis (QDA). Comparisons of average posterior/prior ratios show that QDA performed best (Turtle: LDA = 1.483, QDA = 1.693; Lissamphibian: LDA: 1.219, QDA = 1.572).

### DFA Methodology Validation

We validate this new methodology by evaluating its performance in two situations: (1) when analyzing DNA data simulated under conditions known to cause phylogenetic inference problems, and (2) when analyzing empirical amino acid data for a challenging phylogenetic question [47].

For the simulation study, we simulated two 8-taxon datasets under conditions that cause standard phylogenetic methods to recover the incorrect phylogeny. Tree topologies were balanced and included four groups of two sister species. On each side of the short innermost branch are two sister groups, one of which has a short subtending branch and one of which has a long subtending branch. Equilibrium GC content was set to 80% for long branches and the sister groups that they subtend, while it was set to 50% for all other branches. Other parameters of the substitution matrices were equal among branches. Each simulated dataset was 1,500 bp in length and consisted of one 1000-bp subset, and one 500-bp subset. The 500-bp partition [(((A1∶0.05,A2∶0.05):0.5,(B1∶0.05,B2∶0.05):0.02):0.05,((C1∶0.05,C2∶0.05):0.02,(D1∶0.05,D2∶0.05):0.5):0.05);] showed larger differences in branch lengths than the other one [(((A1∶0.05,A2∶0.05):0.3,(B1∶0.05,B2∶0.05):0.02):0.05,((C1∶0.05,C2∶0.05):0.02,(D1∶0.05,D2∶0.05):0.3):0.05);]. Simulations were performed using bppseqgen [73]. Precise command lines used for simulation can be obtained from the authors.

The simulated dataset was filtered for biased sites with DFA comparing the true topology used in simulations and the biased topology in which clades with long branches are clustered together. We used either two descriptive statistics (%GC and site-rates) or one descriptive statistic (site-rate), removing 10%, 20%, 30%, 40% and 50% of the sites. These alignments were compared to the random removal of a comparable number of sites. Phylogenetic analyses of the datasets were performed using RAxML with the same parameters as above. Results of these analyses are found in Table S1. Removing sites according to the strength of DFA prediction has different effects on the phylogenetic inference compared to removing sites at random: DFA-based removal allows recovery of the correct topology when enough sites are removed, but random removal does not. In this simulation, filtering based on DFA prediction excluding %GC performs better at escaping the incorrect topology and closely estimating the true phylogeny (Table S1). This result motivated us to try DFA filtering without clade-specific %GC for our empirical data (see above).

For an empirical test of our methodology, we focus on the Coelomata-Ecdysozoa debate regarding metazoan phylogeny [48]. Although some studies support the traditional Coelomata relationship, evidence is mounting in support of a monophyletic Ecdysozoa (see [49] for a summary). In the multi-gene dataset of Wolf et al. (2003) [47], eight macromolecular complex subunits were analyzed, with 5 of 8 genes supporting the Coelomata relationship. We employ our DFA methodology on each of the eight datasets, with some slight modifications; datasets are comprised of amino acid sequences so we only use site-rates as a filtering variable (no missing data or GC content). We generated five filtered alignments varying in the amount of data removed (10%, 20%, 30%, 40%, 50%), and compare these results to random removal of sites. Results dramatically changed compared to those in the original study [47]; originally 5/8 datasets supported the Coelomata relationship, while after data filtering, 6/8 datasets support the Ecdysozoa relationship (Table S2). Three datasets, based on original analyses and random removal of sites, that support the Coelomata relationship (CH, CL, LF) shift support to the Ecdysozoa relationship after DFA data filtering, while three datasets originally supporting the Ecdysozoa (RI, RP) maintain support after data filtering. One of the datasets originally supporting Ecdysozoa (PR) maintains support after some filtering, but show inconsistent results for the 40% and 50% dataset (neither supporting Coelomate nor Ecdysozoa).

While we endeavored to include those predictor variables that we felt were most likely to be correlated with biased signal in the data, we note that these decisions were subjective and we may have left out stronger correlates. Similarly, interactions among predictors were ignored for the sake of tractability. The potential also exists for true phylogenetic signal to manifest itself in %GC in some cases, leading to the exclusion of sites with unbiased signal. However, for both analyses we repeated the QDA procedure after excluding all %GC variables as predictors and report those results as well. Examining the sensitivity of phylogenetic conclusions to these considerations will be an interesting avenue for future work.

## Supporting Information

Figure S1 **Maximum Likelihood phylogenies of the different data transformations.** Phylogenies have been simplified to only show higher-level relationships within vertebrates. A) All taxa-NUCL dataset, B) 16 taxa-NUCL dataset, C) All taxa-N12 dataset, D) 16 taxa-N12 dataset (note: the Bayesian analysis recovered a turtle-crocodile relationship), E) All taxa-DEGEN1 dataset, F) 16 taxa-DEGEN1 dataset, G) All taxa-AA dataset, H)16 taxa-AA dataset. Support values for phylogenies with all taxa (A,C,E,G) show RAxML bootstrap values only if ≥50. Support values for phylogenies with 16 taxa show support values in the form of RAxML bootstrap/Bayesian posterior probability. An * indicates full support.
(PDF)

Figure S2 **Frequency histogram of the rate of evolution for the 75 molecular markers.** Rate of evolution of the 75 markers was estimated by computing average branch length. The red, vertical line indicates the top 25% fastest genes, which were removed for subsequent phylogenetic analyses.
(PDF)

Figure S3 **Permutation test results for the predictive ability of discriminant function analysis (DFA).** Permutation results are compared to random expectations regarding A) lissamphibian relationships and B) the phylogenetic position of turtles. Preferred lissamphibian relationships were permuted among sites (1,000 replicates). Values on the x-axis are the posterior/prior ratio for the preferred topology averaged across all sites for each replicate. The arrow indicates the empirical value, which falls far outside the null distribution.
(PDF)

Table S1 **Test of discriminant function analysis (DFA) filtering method on simulated data.**
(DOCX)

Table S2 **Test of discriminant function analysis filtering method on empirical example.**
(DOCX)

Table S3 **Predictive power of discriminant function analyses (DFA) for alternative hypotheses.**
(DOCX)

Table S4 **Phylogenetic results from filtered datasets.**
(DOCX)

Table S5 **List of Taxa and Genbank numbers.**
(XLS)

## References

[1] Darwin C (1859) On the origin of species. London: John Murray. 502p.

[2] Yates TL, Salazar-Bravo J, Dragoo JW (2004) The importance of the tree of life to society. In: Cracraft J, Donoghue MJ, editors. Assembling the tree of life. New York: Oxford University Press. 7–17.

[3] Benton MJ (1990) Phylogeny of the major tetrapod groups: morphological data and divergence dates. J Mol Evol 30: 409–424. 2111854 10.1007/BF02101113

[4] Meyer A, Zardoya R (2003) Recent advances in the (molecular) phylogeny of vertebrates. Annu Rev Ecol Evol and Syst 34: 311–338.

[5] Thomson RC, Shaffer HB (2010) Rapid progress on the vertebrate tree of life. BMC Biol 8: 19. 20211001 10.1186/1741-7007-8-19 PMC2842240

[6] Gaffney ES (1980) Phylogenetic relationships of the major groups of amniotes. In: Panchen AL, editor. The terrestrial environment and the origin of land vertebrates. New York: Academic Press. 593–610.

[7] Lyson TR, et al (2011) MicroRNAs support a turtle + lizard clade. Biol Lett 8: 104–107. 21775315 10.1098/rsbl.2011.0477 PMC3259949

[8] Crawford NG, Faircloth BC, McCormack JE, Brumfield RT, Winker K, et al.. (2012) More than 1000 ultraconserved elements provide evidence that turtles are the sister group of archosaurs. Biol Lett. doi:10.1098/rsbl.2012.0331. 10.1098/rsbl.2012.0331 PMC3440978 22593086

[9] Chiari Y, Cahais V, Galtier N, Delsuc F (2012) Phylogenomic analyses support the position of turtles as the sister group of birds and crocodiles (Archosauria). BMC Biol 10: 65. 22839781 10.1186/1741-7007-10-65 PMC3473239

[10] Parsons TS, Williams EE (1963) The relationship of the modern Amphibia: a re-examination. Q Rev Biol 38: 26–53.

[11] Carroll RL (2009) The Rise of Amphibians. Baltimore: The Johns Hopkins University Press. 360p.

[12] Carroll RL (2001) The origin and early radiation of terrestrial vertebrates. J Palaeontol 75: 1202–1213.

[13] Cannatella DC, Vieites DR, Zhang P, Wake MH, Wake DB (2009) Amphibians (Lissamphibia). In: Hedges SB, Kumar S, editors. The timetree of life. New York: Oxford University Press. 353–356.

[14] Vallin G, Laurin M (2004) Cranial morphology and affinities of Microbrachis, and a reappraisal of the phylogeny and lifestyle of the first amphibians. J Vert Paleontol 24: 56–72.

[15] Hedges SB, Maxson LR (1993) A molecular perspective on lissamphibian phylogeny. Herpetol Monogr 7: 27–42.

[16] Feller AE, Hedges SB (1998) Molecular evidence for the early history of living amphibians. Mol Phylogenet Evol 9: 509–516. 9667999 10.1006/mpev.1998.0500

[17] Milner AR (1993) The Paleozoic relatives of lissamphibians. Herpetol Monogr 6: 8–27.

[18] Trueb L, Cloutier R (1991) Toward an understanding of the amphibians: two centuries of systematic history. In: Schultze H-P, Trueb L, editors. Origins of the higher groups of tetrapods: controversy and consensus. Ithaca: Cornell University Press. 233–313.

[19] Ruta M, Coates MI, Quicke DLJ (2003) Early tetrapod relationships revisited. Biol Rev 78: 251–345. 12803423 10.1017/s1464793102006103

[20] Zardoya R, Meyer A (2001) On the origin and phylogenetic relationships among living amphibians. Proc Natl Acad Sci USA 98: 7380–7383. 11390961 10.1073/pnas.111455498 PMC34677

[21] Hugall AF, Foster R, Lee MSY (2007) Calibration choice, rate smoothing, and the pattern of tetrapod diversification according to the long nuclear gene RAG-1. Syst Biol 56: 543–563. 17654361 10.1080/10635150701477825

[22] Roleants K, et al (2007) Global patterns of diversification in the history of modern amphibians. Proc Natl Acad Sci USA 104: 887–892. 17213318 10.1073/pnas.0608378104 PMC1783409

[23] San Mauro D, Vences M, Alcobendas M, Zardoya R, Meyer A (2005) Initial diversification of living amphibians predated the breakup of Pangaea. Am Nat 165: 590–599. 15795855 10.1086/429523

[24] San Mauro D (2010) A multilocus timescale for the origin of extant amphibians. Mol Phylogenet Evol 56: 554–561. 20399871 10.1016/j.ympev.2010.04.019

[25] Anderson JS, Reisz RR, Scott D, Frobisch NB, Sumida SS (2008) A stem batrachian from the Early Permian of Texas and the origin of frogs and salamanders. Nature 453: 515–518. 18497824 10.1038/nature06865

[26] Anderson JS (2008) Focal review: the origin(s) of modern amphibians. Evol Biol 35: 231–247.

[27] Carroll RL (2007) The Palaeozoic ancestry of salamanders, frogs, and caecilians. Zool J Linn Soc 150 (suppl. 1)1–142.

[28] Carroll RL, Holmes R (1980) The skull and jaw musculature as guides to the ancestry of salamanders. Zool J Linn Soc 68: 1–40.

[29] Carroll RL, Boisvert C, Bolt J, Green DM, Philip N, et al.. (2004) Changing patterns of ontogeny from osteopiform fish through Permian tetrapods as a guide to the early evolution of land vertebrates. In: Arratia G, Wilson MHV, Coutier R, editors. Recent advances in the origin and early radiation of vertebrates. Munchen: Verlag Dr. Friedrich Pfeil, Munchen. 321–343.

[30] Li C, Wu X, Rieppel O, Wang L, Zhao L (2008) An ancestral turtle from the Late Triassic of southwestern China. Nature 456: 497–501. 19037315 10.1038/nature07533

[31] Philippe H, Brinkmann H, Lavrov DV, Littlewood TJ, Manuel M, et al (2011) Solving difficult phylogenetic questions: why more sequences are not enough. PLoS Biol 9: e1000602. 21423652 10.1371/journal.pbio.1000602 PMC3057953

[32] Jeffroy O, Brinkmann H, Delsuc F, Philippe H (2006) Phylogenomics: the beginning of incongruence? Trends Genet 22: 225–231. 16490279 10.1016/j.tig.2006.02.003

[33] Pattengale ND, Swenson KM, Moret BME (2010) Uncovering hidden phylogenetic consensus. Bioinformat Res Appl 6053: 128–139. 10.1109/TCBB.2011.28 21301032

[34] Meyer A (1994) Shortcomings of the cytochrome b gene as a molecular marker. T Ecol Evol 9: 278–280. 10.1016/0169-5347(94)90028-0 21236853

[35] Simon C, Buckley TR, Frati F, Stewart JB, Beckenbach AT (1994) Evolution, weighting, and phylogenetic utility of mitochondrial gene sequences and a compilation of conserved polymerase chain reaction primers. Ann Entomol Soc Am 87: 651–701.

[36] Edwards SV, Arctander P, Wilson AC (1991) Mitochondrial resolution of a deep branch in the genealogical tree for perching birds. Proc R Soc Lond B 243: 99–107. 10.1098/rspb.1991.0017 1676522

[37] Blouin MS, Yowell CA, Courtnely CH, Dame JB (1998) Substitution bias, rapid saturation, and the use of mtDNA for nematode systematics. Mol Biol Evol 15: 1719–1727. 9866206 10.1093/oxfordjournals.molbev.a025898

[38] Regier JC, Schultz JW, Zwick A, Hussey A, Ball B, et al (2010) Arthropod relationships revealed by phylogenomic analysis of nuclear protein-coding sequences. Nature 463: 1079–1083. 20147900 10.1038/nature08742

[39] Brinkmann H, Philippe H (1999) Archaea sister group of bacteria? Indications from tree reconstruction artifacts in ancient phylogenies. Mol Biol Evol 16: 817–825. 10368959 10.1093/oxfordjournals.molbev.a026166

[40] Philippe H, Lartillot N, Brinkmann H (2005) Multigene analyses of bilaterian animals corroborate the monophyly of Ecdysozoa, Lophotrochozoa, and Protostomia. Mol Biol Evol 22: 1246–1253. 15703236 10.1093/molbev/msi111

[41] Fong JJ, Fujita MK (2011) Evaluating phylogenetic informativeness and data-type usage for new protein-coding genes across Vertebrata. Mol Phylogenet Evol 61: 300–307. 21742044 10.1016/j.ympev.2011.06.016

[42] Edwards SV, Liu L, Pearl DK (2007) High-resolution species trees without concatenation. Proc Natl Acad Sci USA 104: 5936–5941. 17392434 10.1073/pnas.0607004104 PMC1851595

[43] Cranston KA, Hurwitz B, Ware D, Stein L, Wing RA (2009) Species trees from highly incongruent gene trees in rice. Syst Biol 58: 489–500. 20525603 10.1093/sysbio/syp054

[44] Shimodaira H (2002) An approximately unbiased test of phylogenetic tree selection. Syst Biol 51: 492–508. 12079646 10.1080/10635150290069913

[45] Rodriguez-Ezpeleta N, Brinkmann H, Roure B, Lartillot N, Lang BF, et al (2007) Detecting and overcoming systematic errors in genome-scale phylogenies. Syst Biol 56: 389–399. 17520503 10.1080/10635150701397643

[46] Lemmon AR, Brown JM, Stanger-Hall K, Lemmon EM (2009) The effect of ambiguous data on phylogenetic estimates obtained by maximum likelihood and Bayesian inference. Syst Biol 58: 130–145. 20525573 10.1093/sysbio/syp017 PMC7539334

[47] Wolf YI, Rogozin IB, Koonin EV (2003) Coelomata and not Ecdysozoa: evidence from genome-wide phylogenetic analysis. Genome Res 14: 29–36. 10.1101/gr.1347404 PMC314272 14707168

[48] Aguinaldo AM, Tubeville JM, Linford LS, Rivera MC, Raff RA, et al (1997) Evidence for a clade of nematodes, arthropods and other moulting animals. Nature 387: 489–493. 9168109 10.1038/387489a0

[49] Telford MJ, Bourlat SJ, Economou A, Papillon D, Rota-Stabelli O (2008) The evolution of the Ecdysozoa. Phil Trans R Soc B 363: 1529–1537. 18192181 10.1098/rstb.2007.2243 PMC2614232

[50] Gauthier J, Padian K (1985) Phylogenetic, functional, and aerodynamic analyses of the origin of birds and their flight. In: Hecht JH, Ostrom GV, Wellnhofer P, editors. The beginnings of birds. Eichstatt: Freunde des Jura-Museum. 185–197.

[51] Bhullar B-A, Bever GS (2009) An archosaur-like laterosphenoid in early turtles (Reptilia: Pantestudines). Breviora 518: 1–11.

[52] Laurin M, Reisz R (1995) A reevaluation of early amniote phylogeny. Zool J Linn Soc 113: 165–223.

[53] Lee MSY (1995) Historical burden in systematics and the interrelationships of ‘parareptiles’. Biol Rev 70: 459–547.

[54] Lee MSY (1996) Correlated progression and the origin of turtles. Nature 379: 812–815.

[55] Nagashima H, Sugahara F, Takechi M, Ericsson R, Kawashima-Ohya Y, et al (2009) Evolution of the turtle body plan by the folding and creation of new muscle connections. Science 325: 193–196. 19590000 10.1126/science.1173826

[56] Yates AM, Warren A (2000) The phylogeny of ‘higher’ temnospondyls (Vertebrata: Choanata) and its implications for the monophyly and origins of the Stereospondyli. Zool J Linn Soc 128: 77–121.

[57] Hedges SB, Poling LL (1999) A molecular phylogeny of reptiles. Science 283: 998–1001. 9974396 10.1126/science.283.5404.998

[58] Hubbard TJP, Aken BL, Ayling S, Ballester B, Beal K, et al (2009) Ensembl 2009. Nucleic Acids Res 37: D690–D697. 19033362 10.1093/nar/gkn828 PMC2686571

[59] Stamatakis A (2006) RAxML-VI-HPC: maximum likelihood-based phylogenetic analyses with thousands of taxa and mixed models. Bioinformatics 22: 2688–2690. 16928733 10.1093/bioinformatics/btl446

[60] Edgar RC (2004) MUSCLE: multiple sequence alignment with high accuracy and high throughput. Nucleic Acids Res 32: 1792–1797. 15034147 10.1093/nar/gkh340 PMC390337

[61] Maddison DR, Maddison WP (2005) MacClade 4: Analysis of phylogeny and character evolution. Version 4.08a. http://macclade.org. 10.1159/000156416 2606395

[62] R Development Core Team (2011). R: a language and environment for statistical computing. R Foundation for Statistical Computing, Vienna, Austria. ISBN 3-900051-07-0. URL: http://www.R-project.org/.

[63] Ronquist F, Huelsenbeck JP (2003) MrBayes 3: Bayesian phylogenetic inference under mixed models. Bioinformatics 19: 1572–1574. 12912839 10.1093/bioinformatics/btg180

[64] Abascal F, Zardoya R, Posada D (2005) ProtTest: Selection of best-fit models of protein evolution. Bioinformatics 21: 2104–2105. 15647292 10.1093/bioinformatics/bti263

[65] Stamatakis A, Hoover P, Rougemont J (2008) A rapid bootstrap algorithm for the RAxML web-servers. Syst Biol 75: 758–771. 10.1080/10635150802429642 18853362

[66] Miller MA, Holder MT, Vos R, Midford PE, Liebowitz T, et al. (2009) The CIPRES Portals. CIPRES. 2009-08-04. URL: http://www.phylo.org/sub_sections/portal. Accessed: 2009-08-04. (Archived by WebCite(r) at http://www.webcitation.org/5imQlJeQa).

[67] Nylander JAA (2004) MrModeltest 2.3. Program distributed by the author. Evolutionary Biology Centre, Uppsala University.

[68] Wilgenbusch JC, Warren DL, Swofford DL (2004) AWTY: A system for graphical exploration of MCMC convergence in Bayesian phylogenetic inference. http://ceb.csit.fsu.edu/awty. 10.1093/bioinformatics/btm388 17766271

[69] Shimodaira H, Hasegawa M (2001) CONSEL: for assessing the confidence of phylogenetic tree selection. Bioinformatics 17: 1246–1247. 11751242 10.1093/bioinformatics/17.12.1246

[70] Charif D, Lobry JR (2007) SeqinR 1.0–2: a contributed package to the R project for statistical computing devoted to biological sequences retrieval and analysis. In: Bastolla U, Porto M, Roman HE, Vendruscolo M, editors. Structural approaches to sequence evolution: molecules, networks, populations. Springer Verlag, New York. 207–232.

[71] Venables WN, Ripley BD (2002) Modern applied statistics with S, 4^th^ edition. New York: Springer. 512p.

[72] Pond SLK, Frost SDW, Muse SV (2005) HyPhy: hypothesis testing using phylogenies. Bioinformatics 21: 676–679. 15509596 10.1093/bioinformatics/bti079

[73] Dutheil J, Boussau B (2008) Non-homogeneous models of sequence evolution in the Bio++ suite of libraries and programs. BMC Evol Biol. 8: 255. 10.1186/1471-2148-8-255 PMC2559849 18808672

