# Supplementary figures and images for: A Phylogenomic Approach to Vertebrate Phylogeny Supports a Turtle-Archosaur Affinity and a Possible Paraphyletic Lissamphibia
Source: PLoS One. 2012 Nov 7;7(11):e48990. doi: 10.1371/journal.pone.0048990 (PMC3492174; doi:10.1371/journal.pone.0048990)

## ALL TAXA

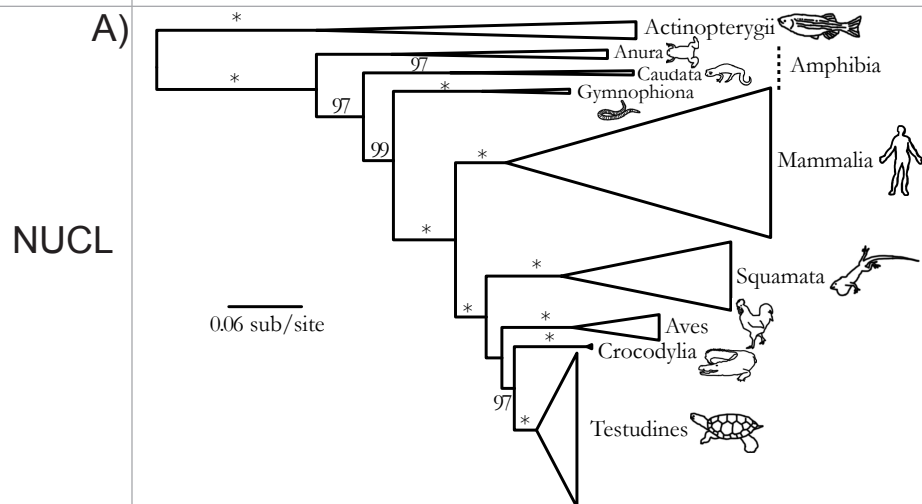

## 16 TAXA

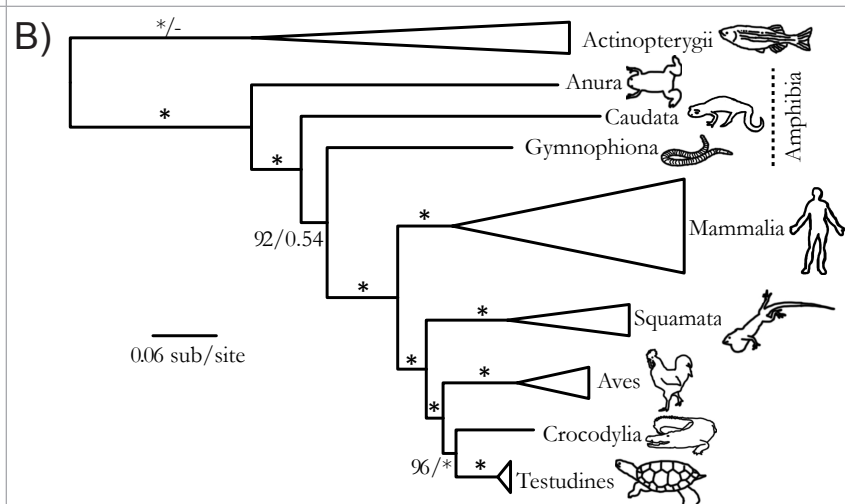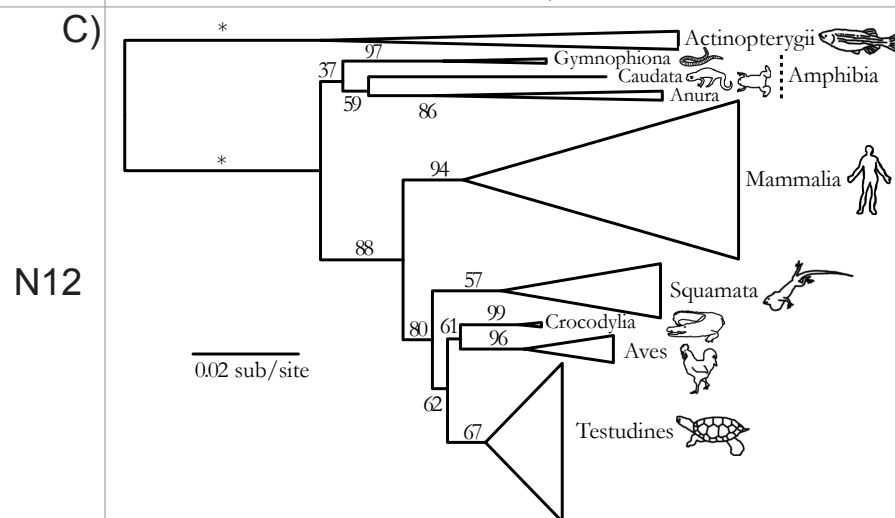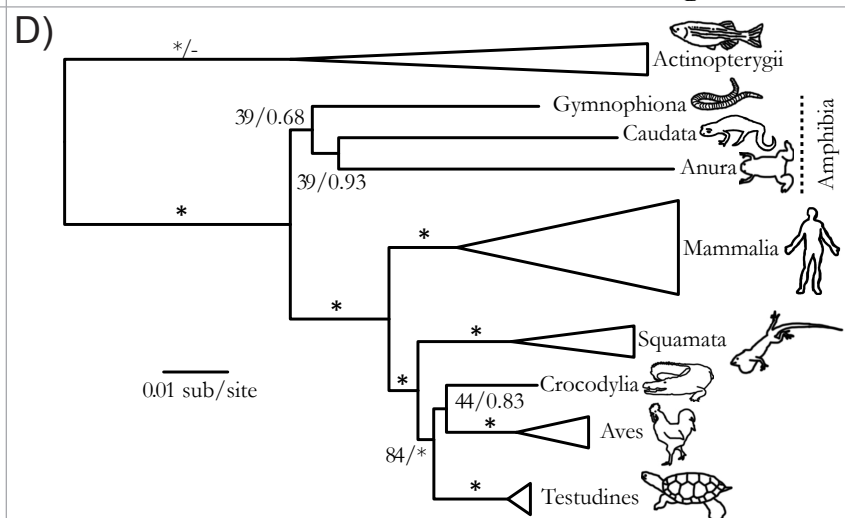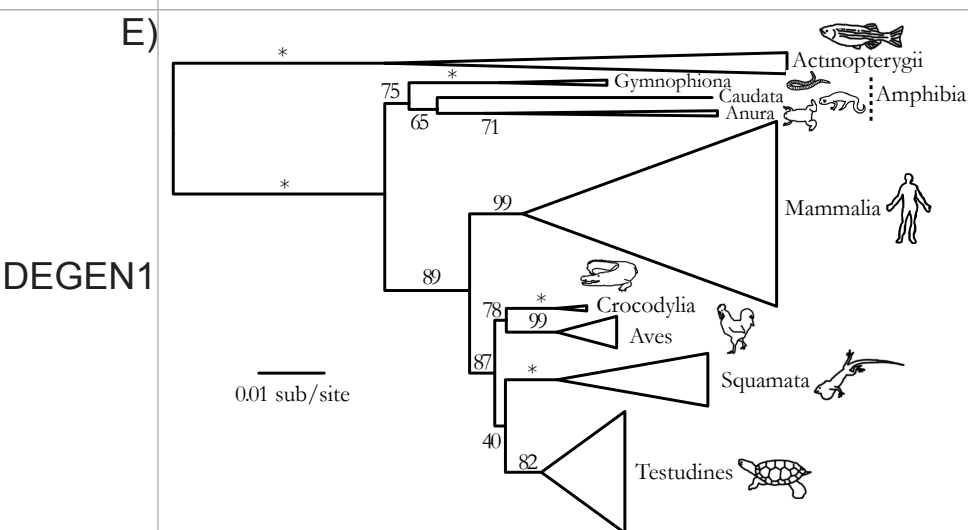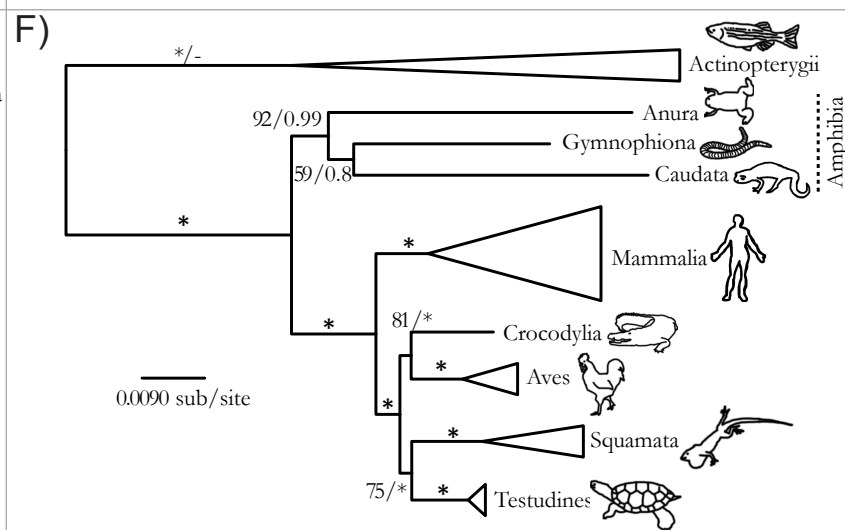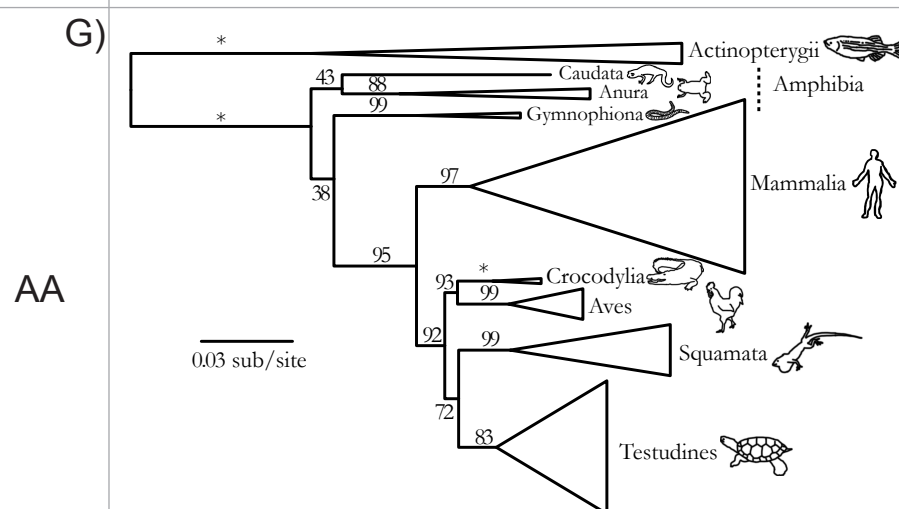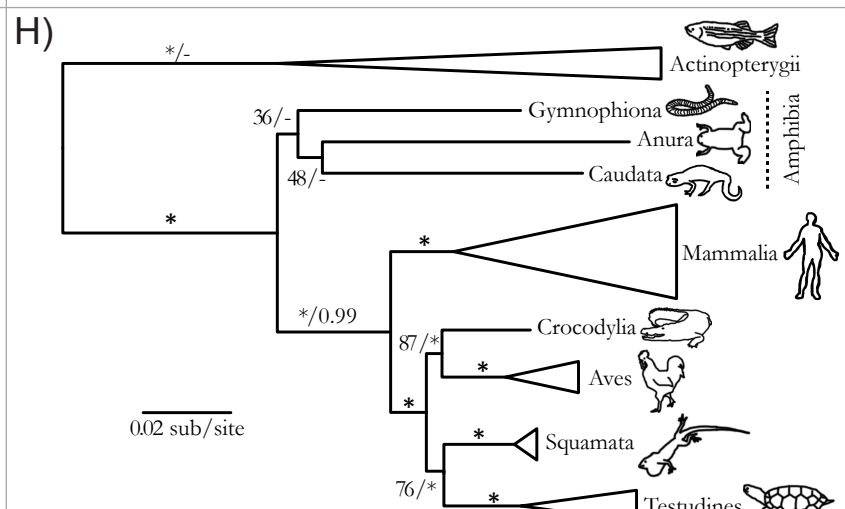

Supplement: Figure S1 — Maximum Likelihood phylogenies of the different data transformations. Phylogenies have been simplified to only show higher-level relationships within vertebrates. A) All taxa-NUCL dataset, B) 16 taxa-NUCL dataset, C) All taxa-N12 dataset, D) 16 taxa-N12 dataset (note: the Bayesian analysis recovered a turtle-crocodile relationship), E) All taxa-DEGEN1 dataset, F) 16 taxa-DEGEN1 dataset, G) All taxa-AA dataset, H)16 taxa-AA dataset. Support values for phylogenies with all taxa (A,C,E,G) show RAxML bootstrap values only if ≥50. Support values for phylogenies with 16 taxa show support values in the form of RAxML bootstrap/Bayesian posterior probability. An * indicates full support. (PDF) [file pone.0048990.s001.pdf]

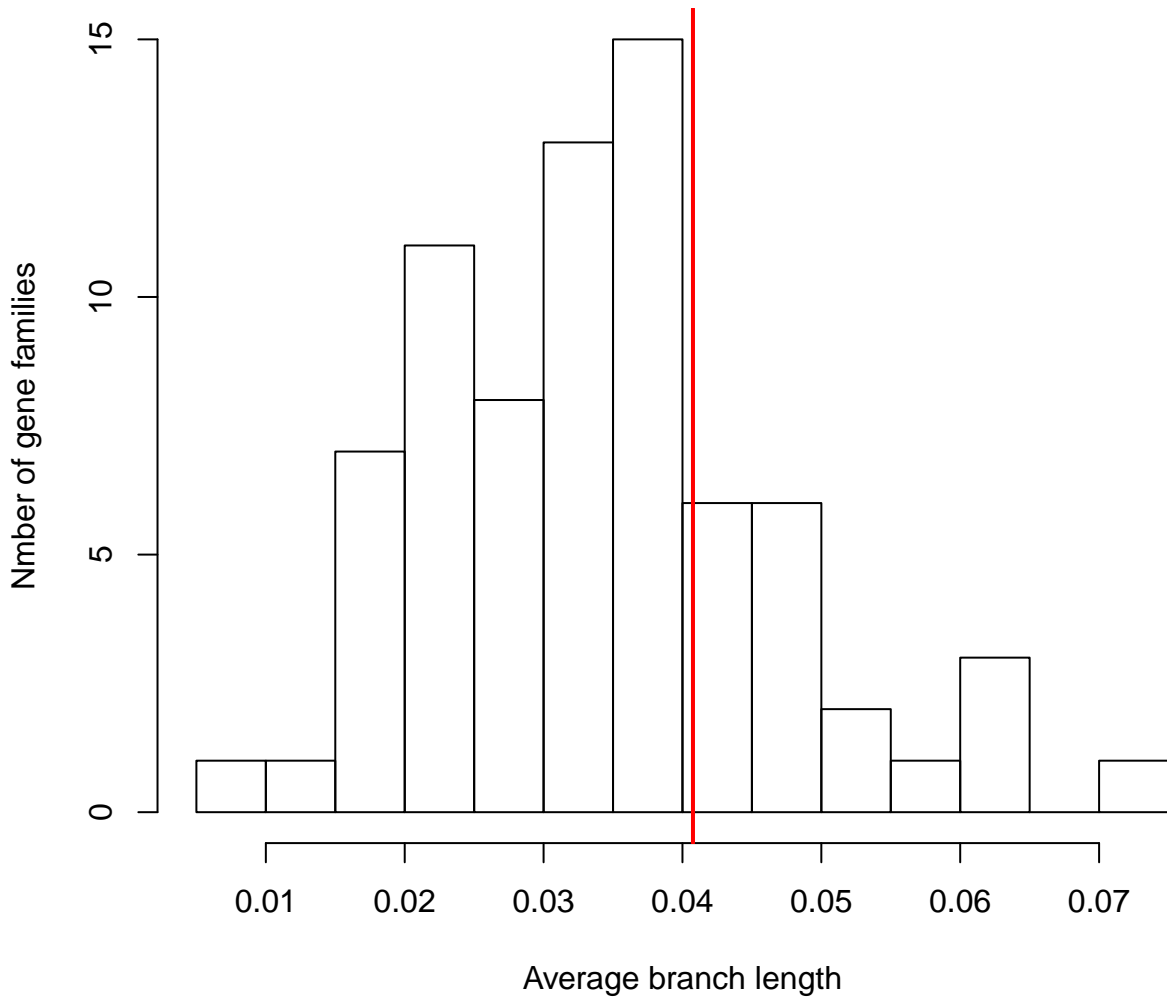

Supplement: Figure S2 — Frequency histogram of the rate of evolution for the 75 molecular markers. Rate of evolution of the 75 markers was estimated by computing average branch length. The red, vertical line indicates the top 25% fastest genes, which were removed for subsequent phylogenetic analyses. (PDF) [file pone.0048990.s002.pdf]

A)

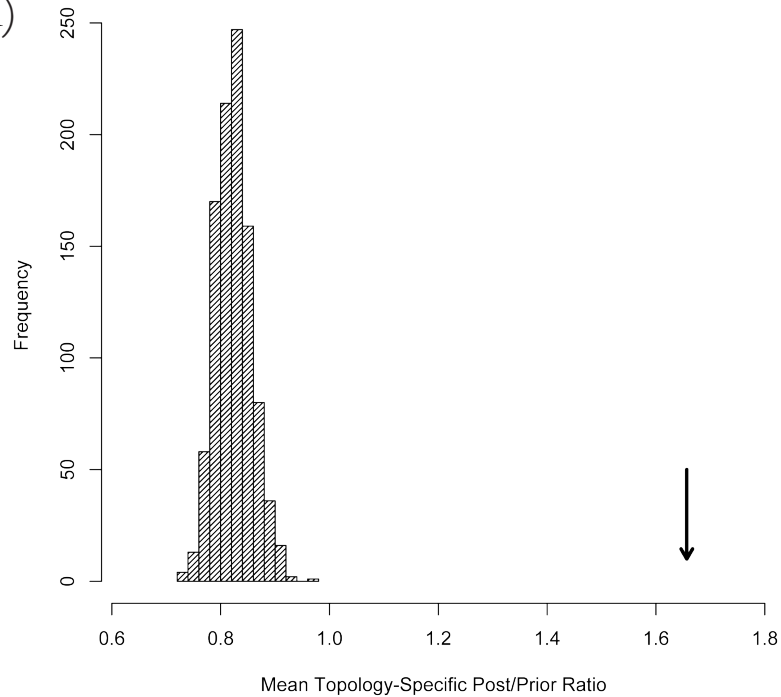

B)

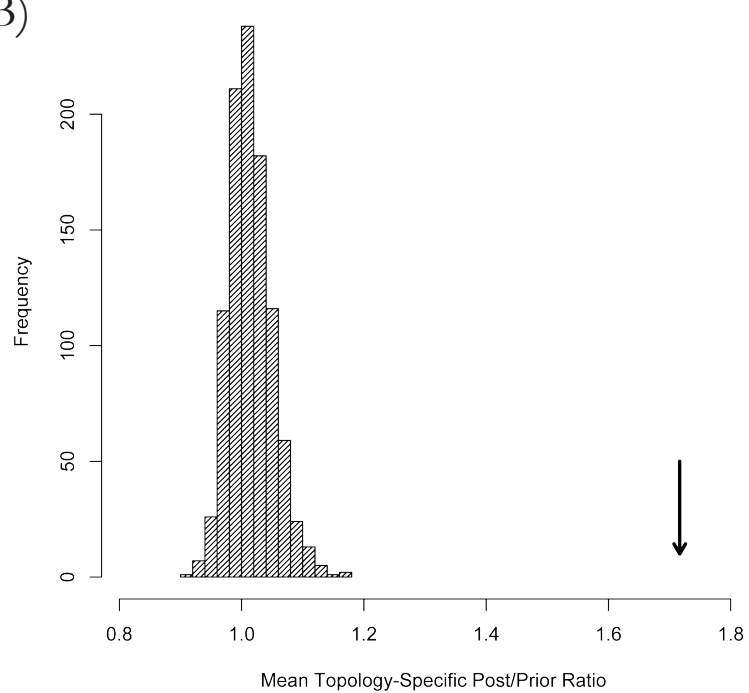

Supplement: Figure S3 — Permutation test results for the predictive ability of discriminant function analysis (DFA). Permutation results are compared to random expectations regarding A) lissamphibian relationships and B) the phylogenetic position of turtles. Preferred lissamphibian relationships were permuted among sites (1,000 replicates). Values on the x-axis are the posterior/prior ratio for the preferred topology averaged across all sites for each replicate. The arrow indicates the empirical value, which falls far outside the null distribution. (PDF) [file pone.0048990.s003.pdf]
