# Supplementary material for: A Phylogenomic Approach to Vertebrate Phylogeny Supports a Turtle-Archosaur Affinity and a Possible Paraphyletic Lissamphibia
Source: PLoS One. 2012 Nov 7;7(11):e48990. doi: 10.1371/journal.pone.0048990 (PMC3492174; doi:10.1371/journal.pone.0048990)
Supplement: Table S1 — Test of discriminant function analysis (DFA) filtering method on simulated data. (DOCX) [file pone.0048990.s004.docx]

|  | 2 types (%GC, site-rates) | | 1 type (site-rate) | |
| --- | --- | --- | --- | --- |
|  | DFA | RANDOM | DFA | RANDOM |
| 10 | (((A1,A2),(D1,D2)),((C1,C2),(B1,B2))) | (((A1,A2),(D1,D2)),((C1,C2),(B1,B2))) | **((((A1,A2),B2),B1),(((D1,D2),C2),C1))** | (((A1,A2),(D1,D2)),((C1,C2),(B1,B2))) |
| 20 | (((A1,A2),(D1,D2)),(((C1,C2),B1),B2)) | (((A1,A2),(D1,D2)),((C1,C2),(B1,B2))) | **((((A1,A2),B2),B1),(((D1,D2),C2),C1))** | (((A1,A2),(D1,D2)),((C1,C2),(B1,B2))) |
| 30 | (((A1,A2),(D1,D2)),(((C1,C2),B1),B2)) | (((A1,A2),(D1,D2)),((C1,C2),(B1,B2))) | **((((A1,A2),B2),B1),(((D1,D2),C2),C1))** | (((A1,A2),(D1,D2)),((C1,C2),(B1,B2))) |
| 40 | (((A1,A2),(D1,D2)),(((C1,C2),B1),B2)) | (((A1,A2),(D1,D2)),((C1,C2),(B1,B2))) | **((((A1,A2),B2),B1),(((D1,D2),C2),C1))** | (((A1,A2),(D1,D2)),((C1,C2),(B1,B2))) |
| 50 | **((((A1,A2),B2),B1),((D1,D2),(C1,C2)))** | (((A1,A2),(D1,D2)),((C1,C2),(B1,B2))) | **((((A1,A2),B2),B1),(((D1,D2),C2),C1))** | (((A1,A2),B2),((C1,C2),(D1,D2)),B1) |

**Table S1. Test of discriminant function analysis (DFA) filtering method on simulated data.**

Results of filtering method with either two types of descriptor variables (%GC and site rates) or one descriptor (site-rates) to remove 10%, 20%, 30%, 40%, or 50% of the data. Results are compared to removal of random sites. Cells with bold font are topologies that are similar or the same as the true toplogy, while cells without bold font are topologies recovered that are similar to or the same as the artifactual topology (incorrect topology.
