# Supplementary material for: A Phylogenomic Approach to Vertebrate Phylogeny Supports a Turtle-Archosaur Affinity and a Possible Paraphyletic Lissamphibia
Source: PLoS One. 2012 Nov 7;7(11):e48990. doi: 10.1371/journal.pone.0048990 (PMC3492174; doi:10.1371/journal.pone.0048990)
Supplement: Table S2 — Test of discriminant function analysis filtering method on empirical example. (DOCX) [file pone.0048990.s005.docx]

| DATASET | **CH** | | **CL** | | DP | | **LF** | | **PR** | | **RI** | | **RP** | | TF | |
| --- | --- | --- | --- | --- | --- | --- | --- | --- | --- | --- | --- | --- | --- | --- | --- | --- |
| Original Result | C (100) | | C (72) | | C (64) | | C (80) | | E (82) | | E (90) | | E (89) | | C (70) | |
|  | DFA | RANDOM | DFA | RANDOM | DFA | RANDOM | DFA | RANDOM | DFA | RANDOM | DFA | RANDOM | DFA | RANDOM | DFA | RANDOM |
| 10 | E (100) | E (73) | C (52) | C (71) | C (97) | C (70) | C (70) | C (54) | E (100) | E (86) | E (100) | E (95) | E (100) | E (77) | C (84) | C (73) |
| 20 | E (99) | C (71) | E (98) | C (43) | C (100) | C (85) | E (86) | C (38) | E (99) | E (93) | E (100) | E (94) | E (100) | E (53) | C (95) | C (66) |
| 30 | E (100) | C (67) | E (100) | C (79) | C (98) | C (79) | E (91) | C (62) | E (99) | E (75) | E (100) | E (75) | E (100) | E (90) | C (90) | C (41) |
| 40 | E (100) | C (49) | E (100) | C (65) | C (99) | E (77) | E (84) | C (95) | X | E (88) | E (100) | E (92) | E (100) | E (74) | C (81) | C (49) |
| 50 | E (97) | C (64) | E (100) | C (43) | C (99) | C (82) | E (83) | C (49) | X | E (83) | E (99) | E (93) | E (96) | E (84) | C (85) | C (37) |

**Table S2. Test of discriminant function analysis filtering method on empirical example.**

Results from testing the DFA on a multi-gene dataset from Wolf et al (2003) using site rate as the sole predictor. The removal of sites using DFA (10-50%) is compared to random removal of sites. In Wolf et al (2003), 5 of 8 genes supported the Coelomata hypothesis, but after DFA filtering method, 6 of 8 genes support the Ecdysozoa hypothesis. C=Coelomata, E=Ecdysozoa, and number in parentheses is the bootstrap support for the topology. Genes in bold font (CH, CL, LF, PR, RI, RP) are those that support the Ecdysozoa hypothesis after DFA filtering.
