# Supplementary material for: A Phylogenomic Approach to Vertebrate Phylogeny Supports a Turtle-Archosaur Affinity and a Possible Paraphyletic Lissamphibia
Source: PLoS One. 2012 Nov 7;7(11):e48990. doi: 10.1371/journal.pone.0048990 (PMC3492174; doi:10.1371/journal.pone.0048990)
Supplement: Table S3 — Predictive power of discriminant function analyses (DFA) for alternative hypotheses. (DOCX) [file pone.0048990.s006.docx]

| **TURTLE** | DFA | Control | DFA/Control |
| --- | --- | --- | --- |
| Archosaur | 0.12 | 0.18 | 0.66 |
| Crocodilian | 0.33 | 0.26 | 1.27 |
| Lepidosaur | 0.69 | 0.19 | 3.62 |
| Basal Sauropsid | 0.15 | 0.10 | 1.52 |
| Basal Amniote | 0.41 | 0.27 | 1.50 |
| **LISSAMPHIBIA** | DFA | Control | DFA/Control |
| Batrachia | 0.31 | 0.21 | 1.43 |
| Procera | 0.24 | 0.12 | 1.98 |
| Paraphyletic  Frog-Salamander | 0.33 | 0.22 | 1.50 |
| Paraphyletic  Caecilian-Salamander | 0.69 | 0.44 | 1.56 |

**Table S3. Predictive power of discriminant function analyses (DFA) for alternative hypotheses.**

Results of DFA for turtle placement and lissamphibian relationships. The DFA uses three variables to predict the alternative topologies: %GC content, % missing data, and site-wise rates of evolution. DFA values are the proportion of sites preferring a particular topology whose support is correctly predicted by DFA. Control values are the expected proportion of sites preferring a particular topology whose support would be correctly predicted using only the control (the control probability of each topology is defined as the frequency of sites preferring that topology). DFA/Control is a standardized measure of the predictive power of the DFA. A value of 1 means the DFA and the control perform similarly as predictors, >1 means the DFA is a better predictor, while <1 means the DFA is a poor predictor. When DFA is a good predictor of a topology, a site’s topological preference can be predicted from only its %GC, %missing, or site-rates.
