# Supplementary material for: A Phylogenomic Approach to Vertebrate Phylogeny Supports a Turtle-Archosaur Affinity and a Possible Paraphyletic Lissamphibia
Source: PLoS One. 2012 Nov 7;7(11):e48990. doi: 10.1371/journal.pone.0048990 (PMC3492174; doi:10.1371/journal.pone.0048990)
Supplement: Table S4 — Phylogenetic results from filtered datasets. (DOCX) [file pone.0048990.s007.docx]

| TURTLE | 3 types (site-rates, %GC, %missing) | | | | | 2 types (site-rates, %missing) | | | | |
| --- | --- | --- | --- | --- | --- | --- | --- | --- | --- | --- |
|  | 50% | 40% | 30% | 20% | 10% | 50% | 40% | 30% | 20% | 10% |
| Archosaur | 7e-5 | 2e-5 | 1e-4 | 1e-4 | 0.031 | **X** | **X** | **X** | **0.313** | **0.173** |
| Crocodilian | **X** | **X** | **X** | **X** | **X** | 0.006 | **0.119** | **0.32** | **X** | **X** |
| Lepidosaur | 6e-79 | 5e-33 | 8e-98 | 2e-54 | 6e-6 | 1e-68 | 1e-7 | 2e-59 | 1e-46 | 1e-48 |
| Basal Sauropsid | 4e-5 | 3e-117 | 4e-5 | 2e-8 | 2e-4 | 8e-65 | 2e-50 | 2e-70 | 1e-56 | 1e-44 |
| Basal Amniote | 6e-5 | 7e-10 | 1e-6 | 1e-84 | 0.001 | 5e-26 | 1e-4 | 5e-7 | 1e-6 | 12e-5 |
| LISSAMPHIBIA | 3 types (site-rates, %GC, %missing) | | | | | 2 types (site-rates, %missing) | | | | |
|  | 50% | 40% | 30% | 20% | 10% | 50% | 40% | 30% | 20% | 10% |
| Batrachia | 7e-36 | 7e-34 | 3e-6 | 2e-65 | 4e-52 | 0.004 | 0.003 | 0.019 | 0.015 | 0.003 |
| Procera | 7e-11 | 5e-61 | 5e-5 | 2e-66 | 93-94 | 0.029 | **0.405** | **0.375** | **0.131** | 3e-4 |
| Paraphyletic  Frog-Salamander | 3e-76 | 2e-7 | 1e-6 | 6e-67 | 5e-6 | 2e-7 | 0.004 | 1e-4 | 0.002 | 0.001 |
| Paraphyletic Caecilian-Salamander | **X** | **X** | **X** | **X** | **X** | 7e-11 | 0.036 | **X** | **X** | **X** |
| Other | - | - | - | - | - | **X** | **X** | - | - | - |

**Table S4. Phylogenetic results from filtered datasets.**

Varying amounts of suspect sites were removed and tested. A) Position of turtles in the amniote phylogeny using three descriptive statistics (site-rates, %GC, and %missing), B) position of turtles in the amniote phylogeny using two descriptive statistics (excluding %GC), C) interrelationships of Lissamphibian groups using three descriptive statistics (site-rates, %GC, and %missing), D) interrelationships of Lissamphibian groups using two descriptive statistics (excluding %GC). The percentage in each column represents the percentage of sites removed from the dataset. Values in cells represent p-values, “X” denotes the best tree, and trees statistically indistinguishable from the best tree are in bold font (Approximately Unbiased topology test p-value > 5%).
